# Enhancement of polyhydroxyalkanoate production by co-feeding lignin derivatives with glycerol in *Pseudomonas putida* KT2440

**DOI:** 10.1186/s13068-020-01861-2

**Published:** 2021-01-07

**Authors:** Zhangyang Xu, Chunmei Pan, Xiaolu Li, Naijia Hao, Tong Zhang, Matthew J. Gaffrey, Yunqiao Pu, John R. Cort, Arthur J. Ragauskas, Wei-Jun Qian, Bin Yang

**Affiliations:** 1 Bioproducts, Sciences & Engineering Laboratory, Department of Biological Systems Engineering, Washington State University, Richland, WA 99354 USA; 2 Department of Chemical and Biomolecular Engineering, University of Tennessee, Knoxville, TN 37996 USA; 3 Biological Sciences Division, Pacific Northwest National Laboratory, Richland, WA 99352 USA; 4 Joint Institute for Biological Sciences, Biosciences Division, Oak Ridge National Laboratory, Oak Ridge, TN 37831 USA; 5 Department of Forestry, Wildlife, and Fisheries, Center for Renewable Carbon, University of Tennessee Institute of Agriculture, Knoxville, TN 37996 USA; 6 College of Food and Bioengineering, Henan University of Animal Husbandry and Economy, Zhengzhou, 450046 Henan China

**Keywords:** Polyhydroxyalkanoate, Lignin derivatives, Glycerol, *Pseudomonas putida*, Co-feeding

## Abstract

**Background:**

Efficient utilization of all available carbons from lignocellulosic biomass is critical for economic efficiency of a bioconversion process to produce renewable bioproducts. However, the metabolic responses that enable *Pseudomonas putida* to utilize mixed carbon sources to generate reducing power and polyhydroxyalkanoate (PHA) remain unclear. Previous research has mainly focused on different fermentation strategies, including the sequential feeding of xylose as the growth stage substrate and octanoic acid as the PHA-producing substrate, feeding glycerol as the sole carbon substrate, and co-feeding of lignin and glucose. This study developed a new strategy—co-feeding glycerol and lignin derivatives such as benzoate, vanillin, and vanillic acid in *Pseudomonas putida* KT2440—for the first time, which simultaneously improved both cell biomass and PHA production.

**Results:**

Co-feeding lignin derivatives (i.e. benzoate, vanillin, and vanillic acid) and glycerol to *P. putida* KT2440 was shown for the first time to simultaneously increase cell dry weight (CDW) by 9.4–16.1% and PHA content by 29.0–63.2%, respectively, compared with feeding glycerol alone. GC–MS results revealed that the addition of lignin derivatives to glycerol decreased the distribution of long-chain monomers (C10 and C12) by 0.4–4.4% and increased the distribution of short-chain monomers (C6 and C8) by 0.8–3.5%. The ^1^H–^13^C HMBC, ^1^H–^13^C HSQC, and ^1^H–^1^H COSY NMR analysis confirmed that the PHA monomers (C6–C14) were produced when glycerol was fed to the bacteria alone or together with lignin derivatives. Moreover, investigation of the glycerol/benzoate/nitrogen ratios showed that benzoate acted as an independent factor in PHA synthesis. Furthermore, ^1^H, ^13^C and ^31^P NMR metabolite analysis and mass spectrometry-based quantitative proteomics measurements suggested that the addition of benzoate stimulated oxidative-stress responses, enhanced glycerol consumption, and altered the intracellular NAD^+^/NADH and NADPH/NADP^+^ ratios by up-regulating the proteins involved in energy generation and storage processes, including the Entner–Doudoroff (ED) pathway, the reductive TCA route, trehalose degradation, fatty acid *β*-oxidation, and PHA biosynthesis.

**Conclusions:**

This work demonstrated an effective co-carbon feeding strategy to improve PHA content/yield and convert lignin derivatives into value-added products in *P. putida* KT2440. Co-feeding lignin break-down products with other carbon sources, such as glycerol, has been demonstrated as an efficient way to utilize biomass to increase PHA production in *P. putida* KT2440. Moreover, the involvement of aromatic degradation favours further lignin utilization, and the combination of proteomics and metabolomics with NMR sheds light on the metabolic and regulatory mechanisms for cellular redox balance and potential genetic targets for a higher biomass carbon conversion efficiency.
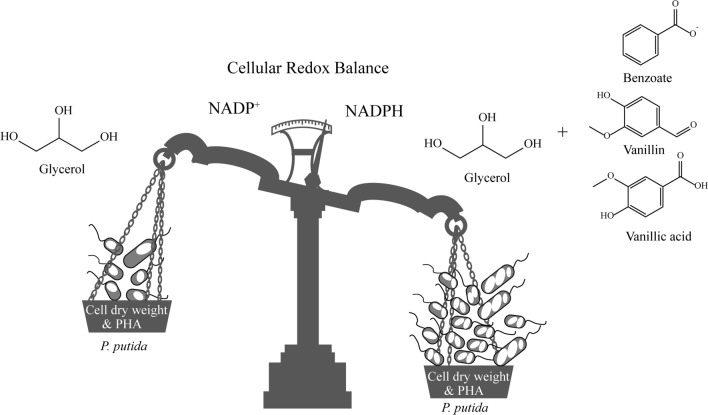

## Background

Current technologies for biomass biological conversion mainly depend on the microbial conversion of biomass-derived sugars to advanced biofuels and bioproducts. However, besides sugars, the efficient utilization of all available carbons from lignocellulosic biomass is critical for economic competitiveness. Unlike photosynthetic systems, where ATP and NADPH are supplied through light reactions, heterotrophic systems must rely on carbon metabolism to produce energy and reductants [1–3]. This is a major limitation for carbon efficiency in biomass conversion into bioproducts via heterotrophic biological systems [4].

As a natural soil resident, *Pseudomonas putida* is capable of divergent carbon substrate utilization, and thrives in versatile nutritional environments [5, 6]. Multiple carbon sources, such as carbohydrates, organics acids, and aromatic compounds can be broken down and transformed into building blocks and energy equivalents for cell growth in *Pseudomonas putida* through cyclic central metabolism pathways, including the Embden–Meyerhof–Parnas (EMP), Entner–Doudoroff (ED), and pentose phosphate (PP) pathways as well as the tricarboxylic acid cycle (TCA) [6–8]. These pathways yield different amounts of NADH/NADPH to meet energy demands, support the biosynthesis of cellular components for the overall cell biomass, and produce bioproducts such as amino acids and lipids [9–11]. *P. putida* attracts significant research attention due to its promising capability to produce medium-chain-length PHA (mcl-PHA) [12] and its consistent availability to genetic manipulation [13]. Polyhydroxyalkanoates (PHA) are a family of intracellular polyesters that accumulate in some bacteria for energy and carbon storage. PHA functions as a reducing equivalent pool to maintain cellular redox balance and defend against oxidative stress (e.g. from nutrients such as N and P being limited or xenobiotic compounds oxidative degradation process such as toluene) through fatty acid synthesis and oxidation process [14–16]. Due to its plastic-like properties and biodegradability, bioplastics made from PHA could potentially replace traditional petroleum plastic. In order to improve PHA biosynthesis, previous studies have mainly focused on nutrient limitation (e.g. N, P, S or Fe) [17], overexpression of PHA synthesis pathways, shutting down competitiveness pathways [18], enhancing the NADH or NADPH supply [19], changing cell growth patterns for rapid proliferation, morphological engineering to increase the cell size [20], the optimization of promoter or the ribosome binding site (RBS) [21], or engineering alternative extremophilic bacteria chassis [22]. In general, the economic competitiveness of PHA production depends on the cost of feedstock, the PHA yield, and the cost of downstream processing. Among these factors, commercial production of PHA is mainly hindered by the cost of the carbon source, which can account for up to 50% of the total cost [23]. In contrast, a large amount of glycerol is generated as a by-product from the increasing production of biodiesel. Glycerol has become an ideal low-cost feedstock for the production of value-added products [24]. Previous studies have extensively investigated the conversion of singular carbon sources into PHA in *P. putida*, including glycerol and the lignin-derived aromatic monomers benzoate, vanillin, and vanillic acid [13, 16, 24, 25]. Thus, *P. putida* can accommodate the catabolism of different types of substrates for both glycerol and lignin-derived monomers. Therefore, using biomass-derived lignin and glycerol to produce PHA is an effective way not only to potentially reduce carbon source costs, but also to increase carbon conversion efficiency.

Co-feeding is a reasonable and inevitable strategy for biomass conversion because after various treatment processes, such as pretreatment and enzymatic hydrolysis, the biomass slurry usually contains mixture compounds, including carbohydrates and lignin polymers, oligomers and monomer sugars, lignin-derived aromatics, and organic acids [26]. Moreover, co-carbon metabolism is an effective and relatively simple approach to potentially decrease the carbon cost and facilitate PHA biosynthesis. The co-feeding of carbohydrates and lignin has been previously investigated in *P. putida* for producing PHA [27, 28]. A multi-omics study revealed that co-feeding glucose and benzoate at a ratio of 1:1 and a total of 100 mM carbon *in P. putida* exhibited an increased growth rate, up-regulated metabolic fluxes in the TCA cycle and benzoate degradation, down-regulated fluxes in glucose oxidation and ED/EMP pathways and yielded a lower quantity of NAD(P)H, compared to feeding glucose alone [5]. Besides, the co-feeding of an organic acid with either carbohydrates or glycerol has been investigated in *P. putida*, including nonanoic acid/glucose with *P. putida* KT2440 and glycerol/octanoate with *P. putida* mt-2 [29, 30]. Simultaneous consumption, higher growth, and higher PHA content were observed under these co-feeding conditions, which indicated that *P. putida* cells assimilate carbon from both the carbohydrate and the organic acid, leading to improved biomass growth and PHA accumulation. These findings indicated that the catabolism of multiple carbon sources can be an alternative way to increase PHA content. Moreover, in the case of co-feeding glycerol with benzene in *P. putida*, the proteins involved in cellular energy metabolism (e.g. glucose-6-phosphate dehydrogenase) were up-regulated, which indicated that the addition of aromatics with glycerol may alter the cellular redox balance in *P. putida* [31]. However, little is known about the underlying cellular physiology and PHA accumulation patterns that come with the co-feeding of glycerol with lignin derivatives in *P. putida*.

Lignin protects plants from microbial and fungal attacks due to its complex polyphenolic polymeric structures [32–34]. The abundance of enzymes available to respond to oxidative stress increases in vivo during lignin catabolism in both bacterial and fungal systems [32, 33, 35]. Thus, biological lignin valorization is a challenging process. The bioconversion of biomass-derived lignin to value-added products by *Pseudomonas putida* has been recently demonstrated [11, 36, 37]. The bioprocessing of low-cost lignin to PHA can potentially reduce costs for both biomass biorefineries and the glycerol-to-bioplastic process. Using aromatic monomers derived from depolymerized lignin is an effective and bottom-up way to simulate the lignin bioconversion process [37, 38]. In previous research*, P. putida* has demonstrated the capacity to convert lignin derivatives (i.e. benzoate, vanillin, and vanillic acid) both with or without glucose into PHA [13, 16, 25, 37]. Data from the bioconversion of lignin model compounds by *P. putida* provides evidence to help decipher and map the lignin bioconversion process and further guide rational genetic manipulation design [25].

In this study, the novel process of co-feeding lignin derivatives and glycerol in *Pseudomonas putida* KT2440 was investigated. The cell growth and PHA content/yield under various co-feeding conditions were determined. Intracellular metabolite analysis and cellular proteomics were conducted to examine glycerol degradation and downstream reducing power generation pathways. Furthermore, reducing equivalent measurements were carried out in order to monitor and cross-examine the NADH and NADPH concentration during the glycerol/lignin derivative co-feeding fermentation process. This study tested a dual-carbon utilization strategy to improve the PHA content/yield and provide new insights into lignin conversion to value-added products.

## Results and discussion

### Cell growth, PHA content, and substrate consumption during co-feeding of aromatic compounds and glycerol to *P. putida* KT2440

*Pseudomonas putida* are natural producers of mcl-PHAs due to their unique metabolic versatility to synthesize polymers when grown on various carbon sources, such as glucose, glycerol, fatty acids, and lignin derivatives [29, 39]. *P. putida* KT2440 exhibits a strong ability to convert lignin derivatives into PHA as a sole carbon and energy source through the *β*-ketoadipate pathway [13, 25, 37]. Herein, the physiological characteristics and mcl-PHA synthesis capabilities of *P. putida* KT2440 were investigated using 10 g L^−1^ glycerol as the main carbon source with the addition of 0.5 g L^−1^ benzoate, vanillin, or vanillic acid as the co-metabolic substrate, with a fixed NH_4_Cl concentration of 0.8 g L^−1^.

**Fig. 1:**
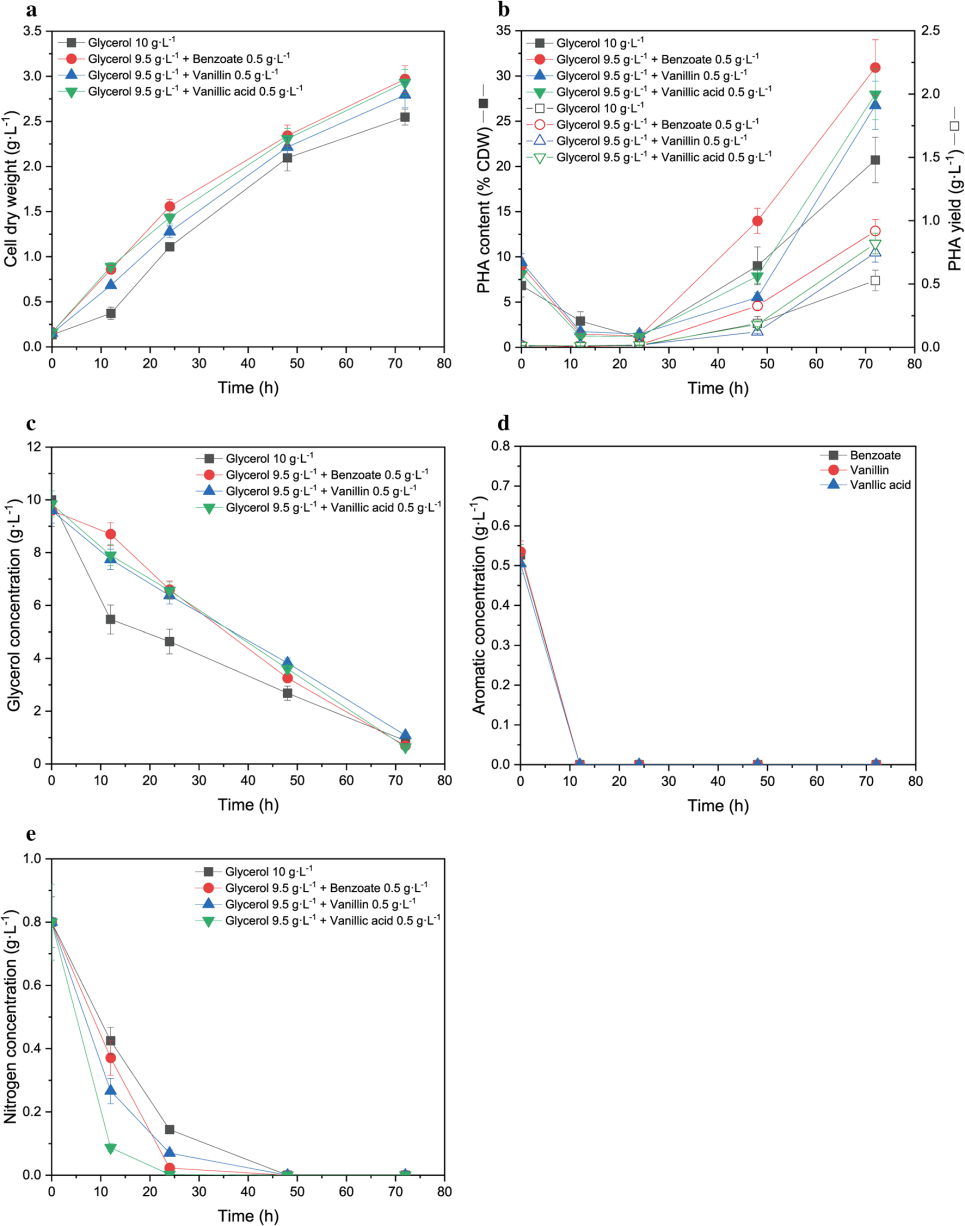
Cell growth and PHA production from co-metabolism of glycerol and aromatic compounds by *P. putida* KT2440. **a** Cell growth, **b** PHA content/yield, **c** glycerol utilization, **d** aromatic compounds utilization, **e** nitrogen source utilization

Figure 1 depicts the cell dry weight (CDW), PHA content/yield, main carbon source consumption, and nitrogen consumption compared to the co-metabolism of glycerol/aromatic compounds over time. As shown in Fig. 1a, with 10 g L^−1^ glycerol alone, cells grew slowly and reached 0.4 g L^−1^ cell dry weight (CDW) at 12 h, followed by rapid growth until 48 h, then gradually reached 2.6 g L^−1^ CDW at 72 h. With the addition of 0.5 g L^−1^ benzoate, vanillin, or vanillic acid with 9.5 g L^−1^ glycerol, the cell growth rapidly increased to 3.0 g L^−1^, 2.8 g L^−1^ and 3.0 g L^−1^ CDW, respectively, at 72 h. Results indicated that the addition of 0.5 g L^−1^ aromatic monomer (i.e. benzoate, vanillin, and vanillic acid) stimulated cell growth as the cell dry weight at 72 h increased by 16.1%, 9.4% and 15.0%, respectively, compared to when feeding glycerol alone.

The same trend was also revealed in PHA content/yield. As shown in Fig. 1b, with 10 g L^−1^ glycerol alone, the PHA content decreased from 6.8% (0.009 g L^−1^) to 2.9% (0.01 g L^−1^) of CDW at 12 h and to 1.1% (0.01 g L^−1^) of CDW at 24 h, then significantly increased to 20.7% of CDW (0.5 g L^−1^) at 72 h. With the addition of 0.5 g L^−1^ of benzoate, vanillin, and vanillic acid, PHA content/yield followed the same declining trend during the first 24 h, and continuously grew rapidly to 30.9% (0.9 g L^−1^), 26.7% (0.7 g L^−1^) and 28.0% (0.8 g L^−1^) of CDW, respectively, at 72 h. Compared to 10 g L^−1^ glycerol alone, the addition of 0.5 g L^−1^ benzoate, vanillin, and vanillic acid with 9.5 g L^−1^ glycerol increased the PHA content by 49.3%, 29.0% and 35.3% of the CDW at 72 h, respectively. Among the three aromatic monomers, benzoate showed the strongest effects in terms of enhancing cell growth and PHA content.

In addition, concomitant assimilation of glycerol, aromatic compounds, and the nitrogen source was observed. Glycerol was rapidly consumed within 72 h, both with and without the addition of aromatic compounds (Fig. 1c), although the addition of aromatic compounds slightly slowed down the glycerol consumption, but did not reduce the extent of its consumption at 72 h. All the aromatic compounds in the co-feeding treatment were nearly completely consumed within the first 12 h (Fig. 1d). With all four different combinations of carbon sources, the nitrogen concentration dramatically declined during the first 24 h and was gradually exhausted by 48 h, while the addition of aromatic compounds appeared to accelerate nitrogen consumption (Fig. 1e). These results suggested that PHA started to accumulate after 24 h, when the nitrogen supply entered a limited level.

Compared to genetic modification, co-feeding of multiple carbon substrates allows direct access to multiple pathways [40] thus may meet the biosynthetic requirements for improved PHA yield [23, 24]. Effects of co-feeding of multiple carbon substrates on PHA synthesis vary depending on the different combinations of carbon sources. Co-feeding glycerol with organic acids (e.g. octanoate) enhanced PHA content to 32.4% of CDW by *P. putida* mt-2, plausibly resulting from increased precursors for cell growth and PHA synthesis with the presence of organic acids [29]. On the contrary, co-feeding aromatic compounds with glycerol showed negative effects on cell growth and PHA synthesis. For example, Kenny et al. [41] reported that co-feeding glycerol with terephthalic acid (TA) to *P. putida* Go16 reduced cell growth and PHA yield when compared to feeding glycerol alone. *Pseudomonas sp.* have been reported to harbour the carbon source preference in the order as organic acid > glucose > aromatic compounds [42]. In the case of co-feeding glucose with aromatic compounds (benzoate, vanillin and vanillic acid) in *P. putida*, aromatic compounds were preferentially utilized over glucose [42–44]. Besides, co-feeding benzoate (0.1%) with glucose (0.25%) showed the highest cell growth compared to co-feeding vanillin (0.1%) or vanillic acid (0.1%) with glucose (0.25%) in *P. putida* CSV86. Similar to our results, co-feeding organic acids (e.g. succinate) with aromatic compounds (e.g. vanillin) showed co-metabolism of dual-carbon sources in *P. putida* CSV86 [42].

Lignin break-down slurries consist of complex mixtures of lignin derivatives. Therefore, using model compounds such as benzoate in this study on co-feeding of lignin derivatives with glycerol simplify the analysing process and enable elucidation of the potential mechanism. Besides, benzoate exhibits similar structure as other lignin derivative compounds and has been used as one of the model compounds for investigating the *β*-ketoadipate pathway, which is very important for lignin break-down products [45, 46]. Moreover, vanillin and vanillic acid were considered as the important intermediate compounds in the funnelling pathways in the lignin bioconversion process [47, 48]. Therefore, the three aromatic compounds (i.e. benzoate, vanillin and vanillic acid) were selected as representatives of the large pool of lignin-derived compounds in this study.

The structural complexity of these aromatic compounds may contribute to their effects on cell growth and PHA accumulation. Ravi et al. [45] reported various lignin derivatives (5 mM) exhibited different effects on cell growth in *P. putida* KT2440 where cells grown on benzoate exhibited the highest growth rate compared with vanillin and vanillate. Although the uptake rate of vanillin was higher than benzoate and vanillic acid in *P. putida*, vanillin enters coniferyl alcohol branch of the upper funnelling pathway, converts vanillin to vanillic acid at the first step of utilization [13, 49]. The utilization of vanillin in *P. putida* was limited to the consumption of vanillic acid. While the consumption rate of vanillic acid was lower than that of benzoate, which might be because the presence of phenylmethyl ether linkage limits the catabolism efficiency [45]. Therefore, higher cell growth with benzoate than that with vanillin or vanillic acid can plausibly be a result of its simpler structure.

Glycerol is the main waste product of biodiesel production through the transesterification of animal fats and vegetable oils [23]. Glycerol has become a promising substrate for PHA production. In addition, lignin is considered as a low-value waste product from biorefinery processes [16]. In this study, the results of co-feeding of glycerol and lignin-derived aromatic compounds, including benzoate, vanillin, and vanillic acid, showed substantial enhancement on cell growth and PHA content compared to feeding glycerol alone. This provides a unique strategy to potentially lower production costs and improve PHA content/yield. The PHA compositions and their underlying mechanisms were further investigated in the following experiments.

### Effects of lignin derivatives on PHA compositions

The GC–MS analysis of the PHA samples from the co-feeding of different aromatic monomers with glycerol confirmed the presence of six monomers, including 3-hydroxyhexanoate (3HHX, C6:0), 3-hydroxyoctanoate (3HO, C8:0), 3-hydroxydecanoate (3HD, C10:0), 3-hydroxydodecanoate (3HDD, C12:0), and 3-hydroxytetradecanoate (3HTD, C14:1/C14:0) (Additional file 1: Figures S1 and S2, Table 1). 3HD (C10:0) was the predominant monomer under all fermentation conditions and accounted for up to 63.3% of the total PHA monomer composition, which was consistent with previous reports [24, 50]. Compared to the PHA grown on glycerol only, with the addition of aromatic compounds (benzoate, vanillin or vanillic acid), the content of longer chain length monomers decreased from 63.3% (glycerol control) to 58.9% (co-feeding glycerol with benzoate) for 3HD (C10:0), 8.3% (glycerol control) to 7.6% (glycerol with vanillic acid) for 3HDD (C12:0), respectively. While the content of shorter chain length monomers increased from 3.5% (glycerol control) to 4.5% (glycerol with either vanillin or vanillic acid) for 3HHX (C6:0) and from 20.4% (glycerol control) to 23.9% (glycerol with vanillic acid) for 3HO (C8:0). Unsaturated and saturated 3HTD (C14:1, C14:0) remained at around 4% and less than 1% under all the tested fermentation conditions. Our results showed that the addition of aromatic compounds decreased the proportion of long-chain monomer (C10:0 and C12:0) of PHA polymer and increased the short-chain monomer (C6:0 and C8:0) proportion. Previous reports indicated that compared to glycerol alone, co-feeding terephthalate (TA) with glycerol also decreased the amount of 3-hydroxydecanoic acid (3HD, C10:0) and 3-hydroxydodecanoic acid (3HDD, C12:0) and increased the amount of 3-hydroxyoctanoic acid (3HO, C10:0) and 3-hydroxyhexanoic acid (3HX, C6:0) [41]. Besides, previous proteomics studies revealed that the *β*-oxidation pathway was up-regulated when *P. putida* was fed solely with vanillic acid or lignin [13]. Therefore, the addition of lignin derivatives might elevate the fatty acid degradation pathway and decrease the number of long-chain monomers (C10 and C12) in favour of producing more reducing equivalents and short-chain monomers (C8 and C6). Thus, it indicates that PHA polymer compositions are influenced by the substrate co-feeding strategy.

**Table 1:** Monomer compositions of PHA accumulated from different carbon sources

	3HHX	3HO	3HD	3HDD	3HTD	
	C6:0	C8:0	C10:0	C12:0	C14:1	C14:0
	mol %	mol %	mol %	mol %	mol %	mol %
Glycerol 10 g L^−1^	3.5 ± 0.3	20.4 ± 1.2	63.3 ± 3.4	8.3 ± 0.7	4.4 ± 0.4	0.1 ± 0.01
Glycerol 9.5 g L^−1^ + benzoate 0.5 g L^−1^	4.3 ± 0.3	23.5 ± 1.3	58.9 ± 3.9	7.8 ± 0.6	4.8 ± 0.3	0.7 ± 0.08
Glycerol 9.5 g L^−1^ + vanillin 0.5 g L^−1^	4.5 ± 0.3	23.3 ± 1.4	59.5 ± 3.5	7.9 ± 0.6	4.3 ± 0.3	0.5 ± 0.04
Glycerol 9.5 g L^−1^ + vanillic acid 0.5 g L^−1^	4.5 ± 0.3	23.9 ± 1.6	59.4 ± 3.2	7.6 ± 0.6	4.2 ± 0.4	0.4 ± 0.03

3-Hydroxyhexanoate (C6:0, 3HHX), 3-hydroxyoctanoate (C8:0, 3HO), 3-hydroxydecanoate (C10:0, 3HD), 3-hydroxydodecanoate (C12:0, 3HDD), 3-hydroxytetradecanoate (C14:1, C14:0, 3HTD) were determined by GC/MS. All monomer compositions were calculated from two replicates (*n* = 2)

HMBC NMR analysis was used to characterize the monomer composition of PHA produced by *P. putida* KT2440 with glycerol alone (Additional file 1: Figure S3a, Table S1) or co-feeding with benzoate (Additional file 1: Figure S3b, Table S1). The HMBC spectra usually contain multiple bond correlations, including two- or three-bond couplings between ^1^H and ^13^C. Several residual one-bond correlation (HSQC-like) peaks were also observed doublets (two peaks in the ^1^H dimension), an artefact of incomplete filtering of one-bond correlations [51]. As shown in Additional file 1: Figure S3a, b, ^1^H resonances at 2.00 and 2.34 ppm are coupled to alkene carbons at 122.9 and 133.9 ppm indicating they are allylic (adjacent to a double bond) CH_2_ groups [52], distinct from the more common ^1^H resonances from CH_2_ groups in the alkyl chains (around 1.26 ppm). The ^13^C signals of 122.9 ppm and 133.9 ppm correspond to the double bond between Δ5 and Δ6 of C14 [39, 52], whose residual one-bond correlations are visible at the left side of Additional file 1: Figure S3b. The proton signal at 5.16 ppm is assigned to the H-3 CH group, along the backbone of the PHA polymer and connecting with the adjacent monomer residue through the ester bond (–C(O)–O–CH–); the carbon of carbonyl group is C-1 of the preceding residue and has a multiple bond correlation H-3. The shoulder peak at 2.46 ppm and 2.55 ppm are the diastereotopic C-2 CH_2_ group and are clearly coupled with the adjacent C-1 CH in the COSY spectrum (Additional file 1: Figure S4a). Consistently, all of the multiple bond couplings of H-2 demonstrated two peaks closely together. The multiple bond couplings of saturated groups such as CH_2_ or CH_3_ are crowded in the upper right corner of the HMBC spectra. The detailed assignments of each monomer were carefully noted in the spectra, cross-validated by COSY NMR (Additional file 1: Figure S4a) and HSQC NMR spectra (Additional file 1: Figure S4b) and listed in Additional file 1: Table S2. The overlapping peaks were assigned to the same value and also corrected as based on previous publications [52–54]. Notably, the assignments of the longer alkyl chains are approximate, and the accurate values may vary due to the overlap in both the proton and carbon dimensions. Moreover, several unassigned peaks remain in the HMBC (notably at δ^1^H 2.28 ppm, with cross peaks to δ^13^C 24.9, 29.6, and 173.2 ppm), HSQC (δ^1^H/^13^C 2.28/31.1 ppm), and COSY spectra; these may correspond to undiscovered components in the PHA polymer. Overall, the HMBC spectra of PHA from cells grown on glycerol alone or glycerol with benzoate take a similar pattern, indicating that the PHA monomer composition is from C6 to C14 and confirming the monomer characterization by GC–MS.

### Effects of the ratio of glycerol to benzoate on cell growth and PHA synthesis

**Fig. 2:**
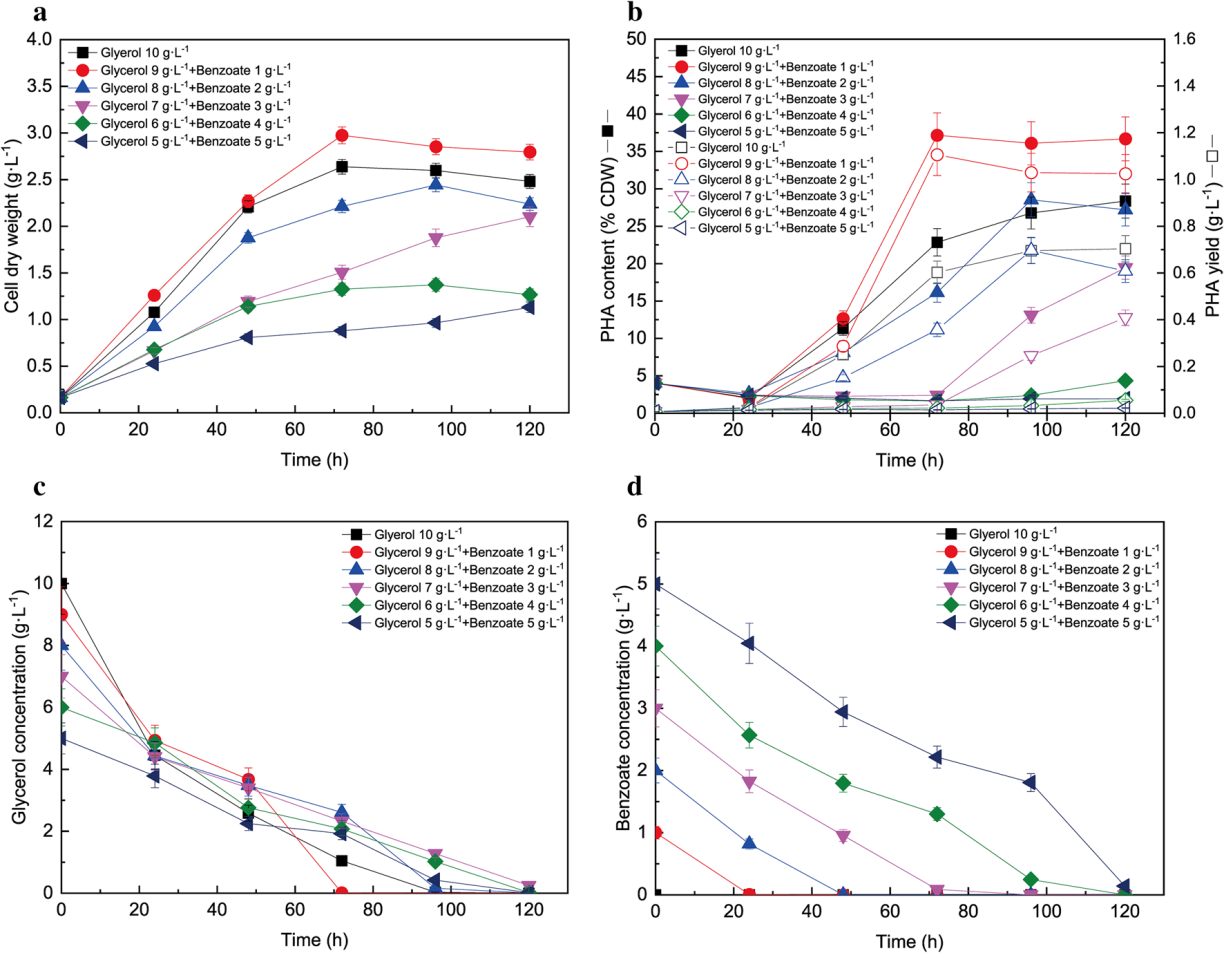
Effects of the ratio of glycerol and benzoate concentration on cell dry weight (**a**), PHA content/yield (**b**) and glycerol utilization (**c**) and benzoate utilization (**d**) by *P. putida* KT2440

To gain more detailed insights into regulation patterns of PHA synthesis, the effects of the ratio of glycerol to benzoate (10 g L^−1^ total carbon source and 0.8 g L^−1^ NH_4_Cl) on CDW (Fig. 2a), PHA content/yield (Fig. 2b), glycerol utilization (Fig. 2c), and aromatic utilization (Fig. 2d) were investigated. The total carbon source was remained at 10 g L^−1^ in order to reduce the effects of fluctuation of carbon supply. As shown in Fig. 2a, for all six combinations of glycerol and benzoate, the cell dry weight generally increased until 72 h and maintained nearly constant until after 120 h total hours of fermentation. Results showed that the addition of 1 g L^−1^ benzoate as a co-substrate with 9 g L^−1^ glycerol led to significant improvement in both cell growth and PHA accumulation compared to feeding glycerol alone. After 72 h of fermentation, compared with 10 g L^−1^ glycerol alone, the CDW and PHA content of *P. putida* KT2440 with 9 g L^−1^ glycerol and 1 g L^−1^ benzoate increased from 2.6 g L^−1^ to 2.9 g L^−1^ (increased by 11.8%) and from 22.8% (0.6 g L^−1^) to 37.2% of CDW (1.1 g L^−1^, increased by 63.2%), respectively. Although the PHA content with 10 g L^−1^ glycerol continuously improved to 28.3% of CDW (0.7 g L^−1^) till 96 h, the PHA content was still lower than that with glycerol 9 g L^−1^ plus benzoate 1 g L^−1^. When the benzoate concentration was higher than 1 g L^−1^, the cell growth and PHA synthesis were repressed. In particular, PHA synthesis nearly ceased and CDW was reduced by more than one-half with feedstocks such as glycerol 6 g L^−1^ + benzoate 4 g L^−1^ or glycerol 5 g L^−1^ + benzoate 5 g L^−1^, although up to 97% of benzoate and glycerol in the medium were consumed. These results indicated that as the benzoate concentration increased to 1 g L^−1^, the PHA content and cell dry weight (CDW) reached the highest value of 37.2% (1.1 g L^−1^) and 2.9 g L^−1^, respectively. Further increased benzoate loading decreased both cell growth and PHA content.

The glycerol concentrations decreased for all six scenarios and reached nearly zero within 120 h. In contrast, the benzoate concentration decreased at a nearly linear consumption rate of 1 g L^−1^ per 24 h for all five scenarios. In addition, co-feeding 1 g L^−1^ benzoate with 9 g L^−1^ glycerol improved cell growth, PHA content, and glycerol consumption when compared to feeding glycerol alone, although the acceleration of glycerol consumption occurred in a delayed manner. These findings indicate that the *P. putida* cells intake carbons from glycerol and aromatic compounds simultaneously in the manner to optimize cell growth [5, 10]. However, the glycerol assimilation was reduced compared to glycerol control under all co-feeding conditions, indicating that the presence of benzoate slightly inhibited the glycerol utilization. Although simultaneous assimilation happened for both glycerol and benzoate in *P. putida* under co-feeding conditions, inhibition still occurred to glycerol utilization, indicating the co-metabolism between glycerol and benzoate was not equally the same. Additionally, during the 48 h to 72 h period, with glycerol 9 g L^−1^ plus 1 g L^−1^ benzoate, the consumption of glycerol was strongly accelerated to reach near-complete consumption at 72 h, faster than all other scenarios, including feeding glycerol alone. This might be due to the dual effects of both benzoate depletion and enhanced cell growth and PHA biosynthesis. In addition, when the initial benzoate was higher than 1 g L^−1^, the resulting cell growth and PHA content were lower than those with feeding glycerol only and concomitant with reduced glycerol assimilation, which might be due to the degradation of PHA and other biomass-derived precursors in order to defend against oxidative stress generated by benzoate [14]. Addition of benzoate and its corresponding oxidative stress generation have been extensively studied in *P. putida* [55–57]. In specific, following adaptation to the presence of aromatic compounds (e.g. benzoate), *P. putida* cells up-regulated the enzymes involved in the detoxification system and antioxidant defence. For instance, proteomics results indicated that enzymes related to detoxification (e.g. ABC transporter) and oxidative stress-related proteins such as catalase/peroxidase were up-regulated when *P. putida* cells fed on benzoate [56, 57]. These findings indicated that the elevated benzoate concentration might maintain the detoxification and antioxidant system activated, and thus the energy demand raised in *P. putida* cells. This caused cells to divert the carbon fluxes to central metabolism and other energy-generating pathways in order to meet the demand, leading to lower cell growth and PHA synthesis fluxes. Thus, whenever the benzoate was nearly consumed and the cell was relieved from the oxidative stress, the PHA synthesis process would then activate as long as the glycerol was still abundant in the medium.

### Effects of nitrogen availability on cell growth and PHA accumulation using glycerol with or without benzoate

Results indicated that the addition of a small amount of benzoate (1 g L^−1^) increased cell growth and PHA accumulation, which suggests that mild oxidative stress might be beneficial. However, the specific mechanisms remain unclear. One possible explanation is the addition of benzoate increased the total equivalent carbon amount, since one molar benzoate is equivalent to seven molars of available carbon and thus increases the carbon and nitrogen ratio. The presence of an appropriate amount of nitrogen source is critical to both cell growth and PHA accumulation. In our previous publication, PHA accumulation rate was reportedly higher under nitrogen-deficient conditions than that under nitrogen-sufficient conditions [25]. Therefore, further investigation is needed to confirm the regulation dependence of PHA synthesis for co-feeding glycerol with benzoate among varied nitrogen source concentrations. Since the amount of benzoate addition did affect PHA synthesis, investigating the relationship between benzoate addition and the nitrogen source would guide future PHA optimization process design. Herein, the effects of benzoate, the nitrogen source and the glycerol ratio on cell growth and PHA accumulation were investigated in this study.

**Fig. 3:**
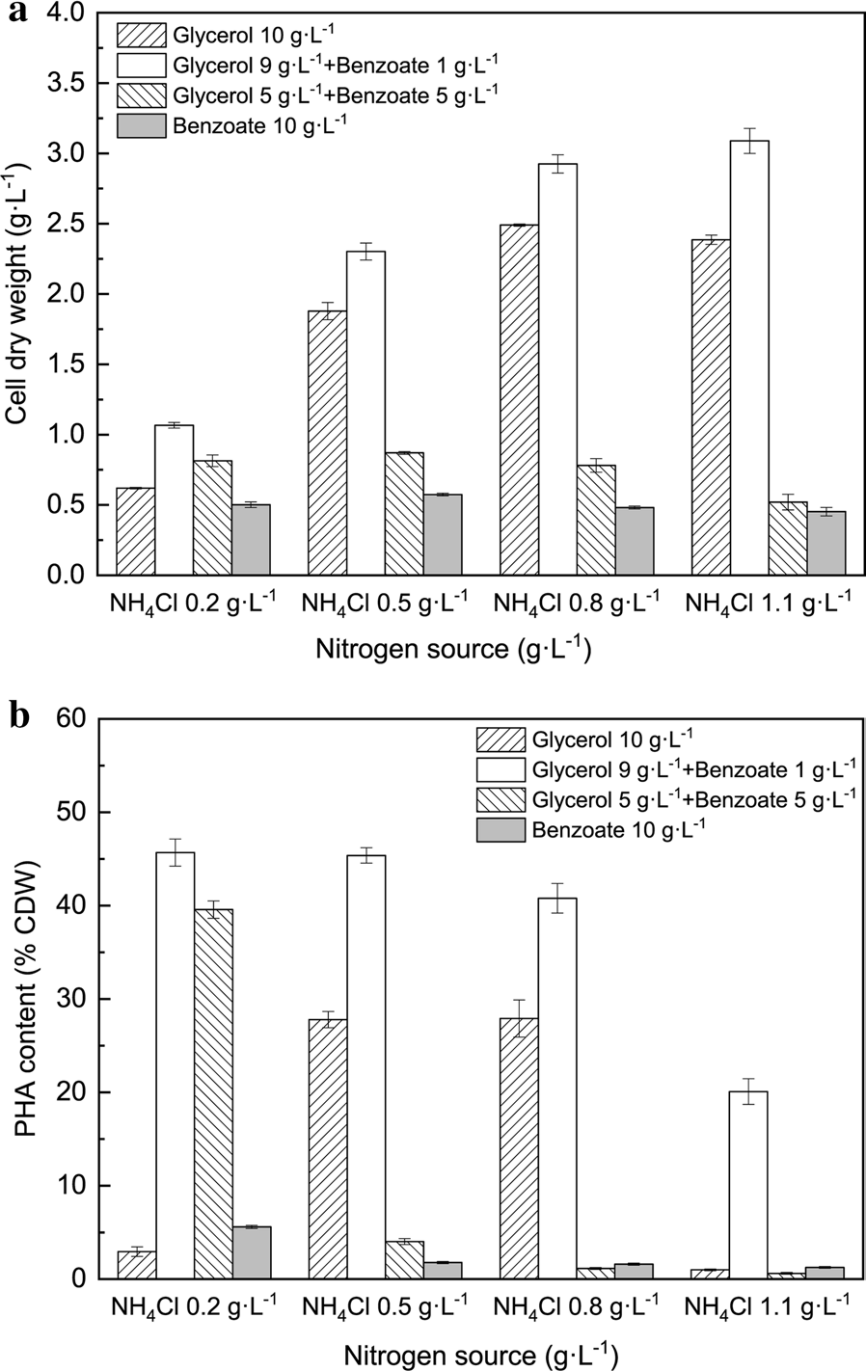
Effects of NH_4_Cl concentrations on cell growth (**a**) and PHA content (**b**) at 72 h by *P. putida* KT2440

Figure 3 shows the effects of NH_4_Cl concentration on the CDW (Fig. 3a) and PHA content (Fig. 3b) using 10 g L^−1^ carbon source. As shown in Fig. 3a, the CDW with 1 g L^−1^ benzoate (8.2 mM) plus 9 g L^−1^ glycerol (97.7 mM, the white column) was higher than that with 10 g L^−1^ glycerol alone under all tested nitrogen concentrations. The CDW with 1 g L^−1^ benzoate plus 9 g L^−1^ glycerol reached the highest amount, at 3.1 g L^−1^ with 1.1 g L^−1^ NH_4_Cl, whereas the CDW only reached 2.4 g L^−1^ with 10 g L^−1^ glycerol alone. Higher benzoate concentrations inhibited cell growth and the CDW was maintained at 0.8 g L^−1^ with 0.2—0.8 g L^−1^ NH_4_Cl and decreased to 0.5 g L^−1^ CDW with 1.1 g L^−1^ NH_4_Cl. When the ratio of benzoate/glycerol was raised to 5/5 and 10 g L^−1^ benzoate alone, cell growth was suppressed and dropped below 0.9 g L^−1^ CDW. As shown in Fig. 3b, when 1 g L^−1^ of benzoate was co-metabolized with 9 g L^−1^ glycerol, the PHA content with various levels of nitrogen supply were all higher than those from 10 g L^−1^ of glycerol. In particular, the maximum PHA content was 45.7% CDW (with 0.2 g L^−1^ NH_4_Cl) and the PHA content decreased as the NH_4_Cl concentration increased. If the benzoate/glycerol ratio changed to 5/5 and 10 g L^−1^ benzoate alone, the PHA content reached its maximum at 39.6% and 5.6% of CDW at 0.2 g L^−1^ NH_4_Cl, respectively, and further decreased significantly as the nitrogen concentration increased. In addition, different timings of nitrogen depletion occurred under different initial nitrogen source conditions, which can affect the cell growth and PHA accumulation. The PHA content using glycerol 9 g L^−1^ plus benzoate 1 g L^−1^ was 1.2 g L^−1^, which was 1.7 times the value achieved from glycerol 10 g L^−1^ with NH_4_Cl at 0.8 g L^−1^. Although the maximum yield of 3.1 g L^−1^ CDW was achieved under the nitrogen-sufficient condition of NH_4_Cl at 1.1 g L^−1^, PHA synthesis was seriously inhibited*. P. putida* KT2440 nearly stopped accumulating PHA when the NH_4_Cl and benzoate concentration exceeded 0.2 g L^−1^ and 5 g L^−1^, respectively. Results showed that even though the addition of benzoate increased the total equivalent carbon amount, the addition of only 1 g L^−1^ benzoate resulted in the highest PHA accumulation content. Theoretically, the PHA content should be further enhanced as the amount of benzoate amount addition increased. However, the results showed that the addition of 9 g L^−1^ glycerol and 1 g L^−1^ benzoate led to the highest cell growth and PHA content at all tested initial nitrogen concentrations. Besides, the cell growth and PHA content both decreased as the benzoate amount was higher than 1 g L^−1^ in spite of different initial glycerol/nitrogen ratios. This result indicated that the effects of benzoate addition on CDW and PHA content were independent from the nitrogen availability, which ruled out the possible correlation of increased carbon-to-nitrogen ratio with PHA synthesis.

### Metabolite profiles and regulatory patterns of glycerol and benzoate co-metabolism in *P. putida* KT2440

PHA is accumulated as carbon and as a reducing power storage polymer in *P. putida* KT2440 under demanding physiological conditions. Maintaining the intracellular redox balance of cells plays a pivotal role in PHA biosynthesis. The pyridine nucleotides NAD(P)^+^ and NAD(P)H are involved in both catabolism and anabolism as the most important redox carriers. Consequently, changes of their intracellular concentrations lead to altered metabolic network patterns [58]. In addition, accurate identification and quantitation of metabolite responses to substrate alteration can provide a detailed physiological characterization of the microorganism and valuable guidance for strategies to enhance PHA synthesis. In this study, NAD(P)^+^/NAD(P)H concentrations, ratios and metabolite profile analysis using NMR were investigated to gain insights on the regulatory patterns of glycerol and benzoate co-metabolism in *P. putida* KT2440.

NMR spectroscopy is a commonly used analytical tool in metabolomics due to its nondestructive, nonbiased, automatable and reproducible features. In addition, by combining one dimensional ^1^H, ^13^C, and ^31^P NMR techniques (Additional file 1: Figures S5–S8, Table S3), NMR metabolomics analysis is particularly effective at detecting and characterizing compounds that are less tractable polar compounds such as sugars, organic acids, phospholipids, and nucleoside compounds (NADP(H)), which mainly compose intracellular metabolite components [59]. In order to investigate the regulatory patterns of the co-feeding strategy of glycerol and benzoate to *P. putida* KT2440, ^1^H NMR spectroscopy was first used to determine extracellular metabolite profiles. Samples were taken at 72 h of fermentation, using glycerol 10 g L^−1^, glycerol 9 g L^−1^ plus benzoate 1 g L^−1^, glycerol 5 g L^−1^ plus benzoate 5 g L^−1^, and benzoate 10 g L^−1^ as carbon sources, respectively (Additional file 1: Figure S5). When feeding with 10 g L^−1^ glycerol alone (Additional file 1: Figure S5a), residue glycerol remained in the media at 72 h. In contrast, the glycerol peaks were barely observed, and no benzoate peaks were detected at 72 h with co-feeding of 9 g L^−1^ glycerol and 1 g L^−1^ benzoate (Additional file 1: Figure S5b), indicating the consumption of glycerol was enhanced, which confirmed the previous HPLC results (Fig. 2c). However, as the benzoate concentration increased to 5 g L^−1^ (Additional file 1: Figure S5c), compared to the other feeding conditions, more glycerol remained in the medium, suggesting that further elevated benzoate concentration suppressed glycerol utilization, which were consistent with the HPLC results before (Fig. 2c, d). Higher peak intensity of benzoate was exhibited in Additional file 1: Figure S5d, reflecting the fact that more benzoate remained unutilized in the medium.

The intracellular metabolites exhibited a distinct profile pattern when compared with the supernatant. Both ^1^H (Additional file 1: Figure S6) and ^13^C NMR spectroscopy (Additional file 1: Figure S7) were used to determine the intracellular metabolite profiles using the same sampling time as the supernatant. Trehalose was clearly identified among all of the feeding conditions and exhibited the highest peak intensity with 10 g L^−1^ glycerol alone (Additional file 1: Figures S6a and S7a). A rapid declining trend of the trehalose signal was observed both in the ^1^H NMR (Additional file 1: Figure S6b–d) and ^13^C NMR spectra (Additional file 1: Figure S7b–d) as the benzoate concentration increased from 1 g L^−1^ to 5 g L^−1^ and maintained a low level when progressing towards 10 g L^−1^. The succinate and acetate compounds stayed at a low level for 10 g L^−1^ glycerol and 9 g L^−1^ glycerol + 1 g L^−1^ benzoate, then spiked to a higher level when the benzoate concentration increased to 5 g L^−1^ and went back to lower levels again for 10 g L^−1^ benzoate (Additional file 1: Figure S6a–d). Acetate acts here as an important intracellular scavenging carbon source for maintaining the acetyl-CoA balance [60, 61]. Our results showed that a 5 g L^−1^ benzoate concentration might inhibit the acetate re-cycling process, which correlates with acetate accumulation. In addition, succinate is the junction between the benzoate degradation pathway and the TCA cycle [46]. An enhanced benzoate concentration might therefore contribute to the observed succinate accumulation. However, too high of a benzoate concentration, such as 10 g L^−1^, might slow down the benzoate utilization due to inhibited cell growth, which was correlated with a lower abundance of succinate and acetate (Additional file 1: Figures S6d and S7d).

In addition, ^31^P NMR was used to characterize the intracellular phosphate-containing extracts (Additional file 1: Figure S8). ^31^P NMR results showed different phosphate profiles when co-feeding glycerol with benzoate at various levels compared to when feeding glycerol or benzoate as the sole carbon source. The signals of NADP^+^ and NADPH were identified among all of the treatments, and it was found that the NADP^+^/NADPH abundance increased in the intracellular extracts as the benzoate concentration increased with the co-feeding of glycerol (Additional file 1: Figure S8a–c). Metabolite profiling using NMR techniques could provide solid results in metabolite characterization. Results suggested that a higher level of PHA synthesis was realized by increasing NADH, NADPH, and glycerol utilization, likely through improved reduction power generation pathways such as the ED pathway and the reduction route of the TCA cycle [9]. It indicated that oxidative stress could manipulate the PHA synthesizing process by altering the intracellular NADPH content, since NADPH is crucial for maintaining antioxidant defences [62]. The increased NADPH can also be diverted to the PHA biosynthesis pathways and the building up of biomass in order to maintain the redox balance of the cell. Reducing equivalent was up-regulated with the addition of benzoate, which might be the directly correlated reason for PHA enhancement. Therefore, cross-evaluation for the intracellular redox state by measuring the NAD^+^/NADH and NADPH/NADP^+^ ratios was needed to further confirm the findings.

**Fig. 4:**
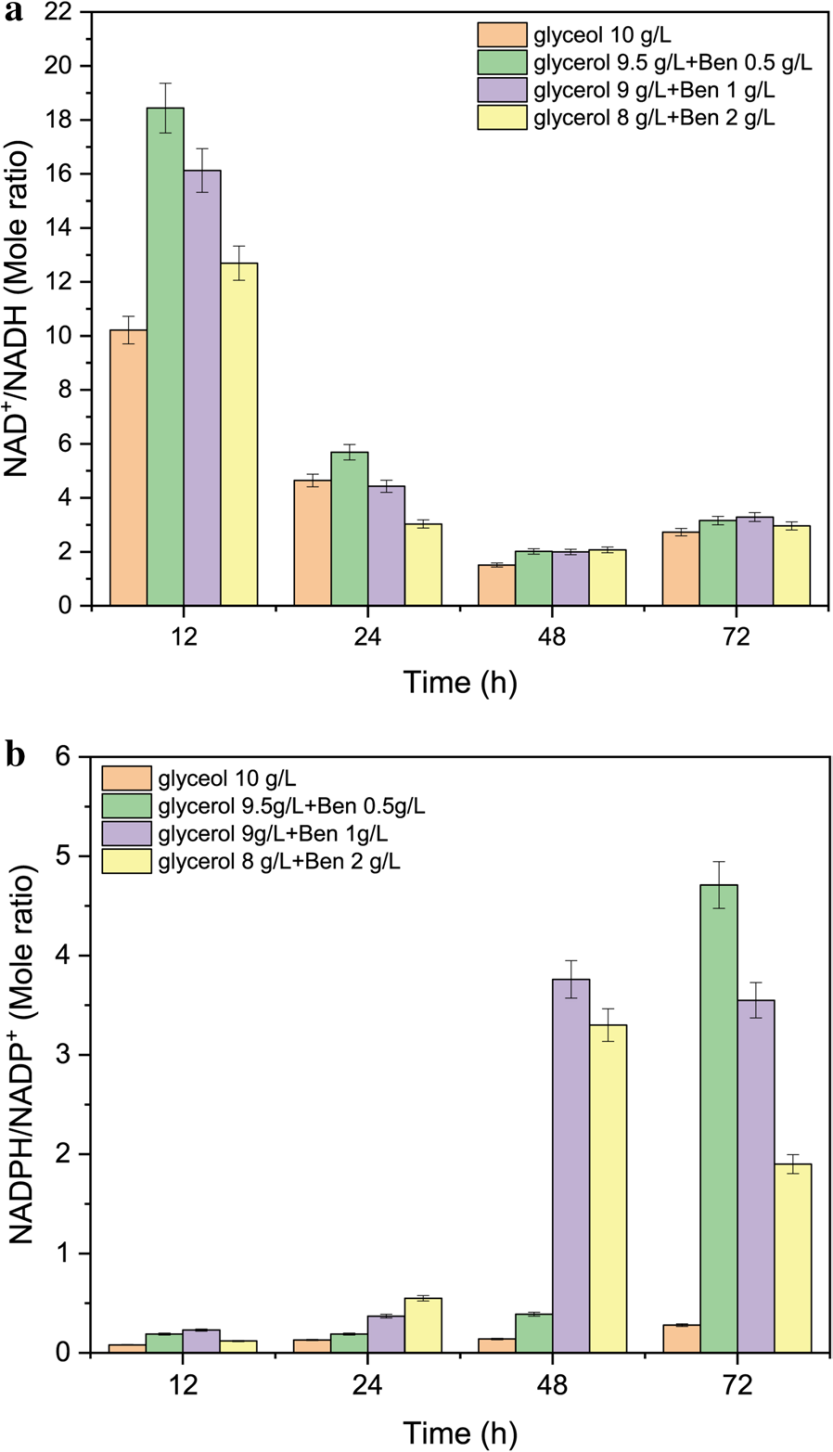
NAD^+^/NADH (**a**) and NADPH/NADP^+^ ratio (**b**) under glycerol and benzoate co-metabolism

In Fig. 4, the intracellular NAD^+^/NADH (Fig. 4a) and NADPH /NADP^+^ ratios (Fig. 4b) over time using different combinations of glycerol and benzoate were elucidated in order to understand the relationship between the redox balance and PHA production. The total pool of NADH was larger than that of NADPH. As shown in Fig. 4a, the ratio of NAD^+^/NADH at 12 h increased dramatically within the test range, especially from 10.2 with glycerol 10 g L^−1^ to 18.4 with glycerol 9.5 g L^−1^ and benzoate 0.5 g L^−1^. As the cultivation time progressed, the ratio of NAD^+^/NADH declined during both the exponential and the late-log phase. For all glycerol/benzoate treatments, the NADH concentration increased during fermentation and reached its highest level at 48 h, followed by a significant decline at 72 h (Additional file 1: Table S4). The NAD^+^/NADH ratio is an important component of the redox state of a cell, and NADH links the TCA cycle to cellular energy generation. The central TCA pathway is regulated by cellular energy level and redox balances, which precisely matches NADH production to respiratory demand. The downward trend in the NAD^+^/NADH ratio and the upward trend in NADH concentration revealed that oxidative stress was strengthened and that the carbon fluxes in the TCA cycle switched to the PHA cycle to some extent by adding a small amount of benzoate (1 g L^−1^ or 0.5 g L^−1^) into the glycerol fermentation system (Figs. 1 and 2). In addition, when adding 2 g L^−1^ benzoate as the co-feeding substrate, the NADH concentration was slightly higher than that with 10 g L^−1^ glycerol as fermentation persisted until 72 h (Additional file 1: Table S4). It is plausible that more benzoate entered the *β*-ketoadipate pathway and converted into succinate and acetyl-CoA, which in turn entered the TCA cycle and produced more NADH in *P. putida* KT2440.

When *P. putida* grew on glycerol alone, the ratio of NADPH/NADP^+^ maintained at low level until 48 h of fermentation and slightly increased at 72 h (Fig. 4b, orange bar). All NADPH/NADP^+^ ratios from the three different combinations of glycerol and benzoate loading dramatically increased after 48 h of fermentation. In specific, with 0.5 g L^−1^ benzoate + 9.5 g L^−1^ glycerol, the ratio of NADPH/NADP^+^ maintained the low level until 24 h, slightly increased at 48 h, and dramatically increased at 72 h of fermentation (Fig. 4b, green bar). Moreover, with 1 g L^−1^ benzoate + 9 g L^−1^ glycerol or 2 g L^−1^ benzoate + 8 g L^−1^ glycerol, the ratio of NADPH/NADP^+^ maintained the low level until 12 h, dramatically increased at 48 h of fermentation and dropped at 72 h of fermentation (Fig. 4b, purple bar and yellow bar). The increased NADPH/NADP^+^ after 48 h of fermentation indicated that the intracellular NADPH concentration strongly increased during the late logarithmic phase in which large amounts of PHA and biomass accumulated. The yields of PHA and CDW using glycerol 9.5 g L^−1^ plus benzoate 0.5 g L^−1^ or glycerol 9 g L^−1^ plus benzoate 1 g L^−1^ were much higher than the values from glycerol 10 g L^−1^ (Figs. 1 and 2). While NADH participates in catabolic reactions, NADPH predominantly acts as a reducing agent in anabolic reactions, such as biosynthesis of PHA biopolymers and active biomass [63]. Therefore, the upward trend of NADPH/NADP^+^ under co-feeding conditions suggested the activation of enzymes involved in reducing power generation pathways, which still needs further confirmation through proteomics analysis.

### Cellular proteome profiles of co-feeding glycerol with benzoate in *P. putida*

In general, bacteria have evolved several strategies to be able to adapt to fluctuating environments to ensure their survival. One of them is up-regulating or down-regulating the synthesis of certain proteins while metabolizing available nutrients [64]. Therefore, to identify potential enzymes and pathways involved in the co-feeding of glycerol and benzoate in *P. putida*, the global proteome profiles of intracellular extracts were analysed.

**Fig. 5:**
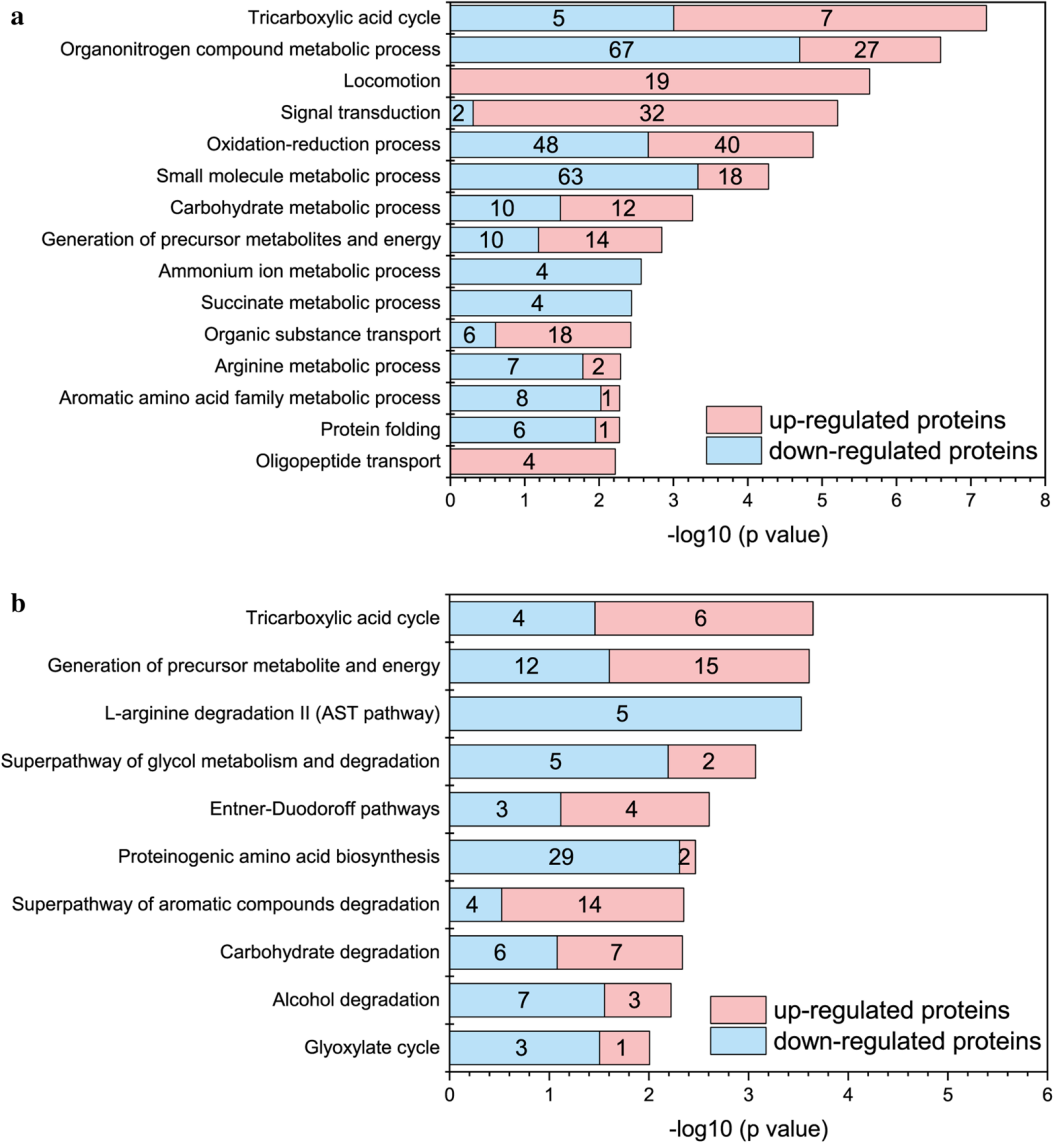
Biological process and pathway enrichment for intracellular proteins in *P. putida*. The enriched biological process (**a**) and enriched KEGG pathways (**b**) between glycerol control (10 g L^−1^) and co-feeding 9 g L^−1^ glycerol with 1 g L^−1^ benzoate conditions. The top 15 enriched biological processes were plotted. The top 10 enriched pathways were also plotted. The bar length corresponds to transformed p value. Numbers in the bar graph indicate the total number of up-regulated or down-regulated proteins in each enriched biological process or KEGG pathway

The top 15 enriched biological processes with a *p* value of less than 0.01 are presented in descending form in Fig. 5a. Notably, the addition of benzoate activates a series of up-regulations of chemotaxis proteins (e.g. chemotaxis histidine kinase CheA, Additional file 2: Table S5) in the signal transduction process of *P. putida*. Moreover, the locomotion process was exclusively up-regulated, demonstrating that *P. putida* activated the biosynthesis of flagellum to facilitate chemotaxis for tumbling and swimming away from the toxic benzoate compound [64, 65]. In addition, eleven out of 15 biological processes contained both up- or down-regulated proteins, indicating that the metabolic pathways were dynamically altered with the addition of benzoate. Therefore, pathway enrichment analysis was further performed to examine the metabolic response that occurs when co-feeding glycerol with benzoate.

**Fig. 6:**
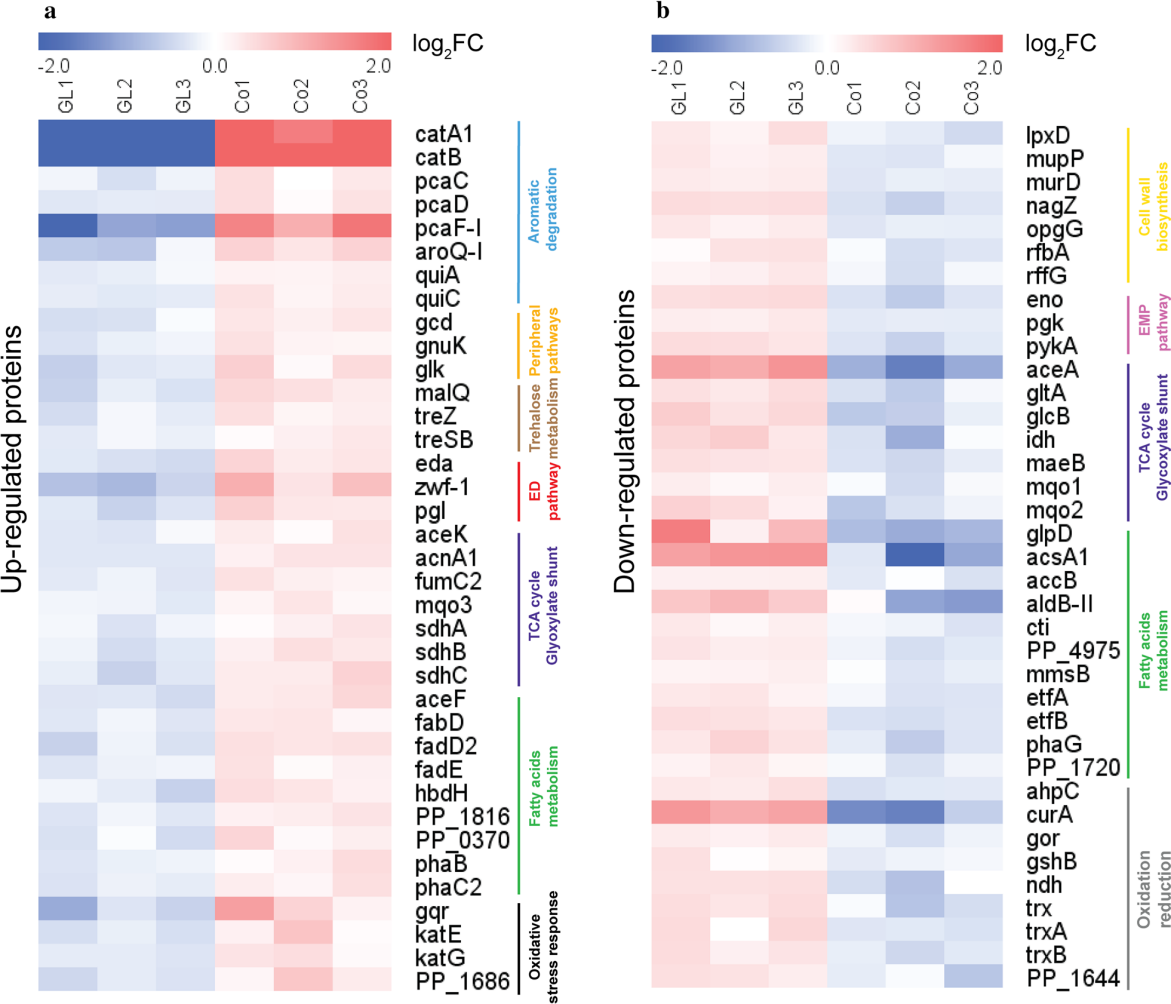
Overview of the up-regulated (**a**) or down-regulated (**b**) proteins (*q* value < 0.05, and fold-change > 1.3) among the intracellular proteomes from glycerol control (10 g L^−1^) or 9 g L^−1^ glycerol + 1 g L^−1^ benzoate fermentation after 3 days. Each row represents one protein and each column represented one sample. Relative abundances were log2 transformed. “GL1”, “GL2” and “GL3” are the triplicate samples from glycerol fermentation; “Co1”, “Co2”, and “Co3” are the triplicate samples from co-feeding glycerol + benzoate. The abbreviation of each protein is listed in Additional file 3

**Fig. 7:**
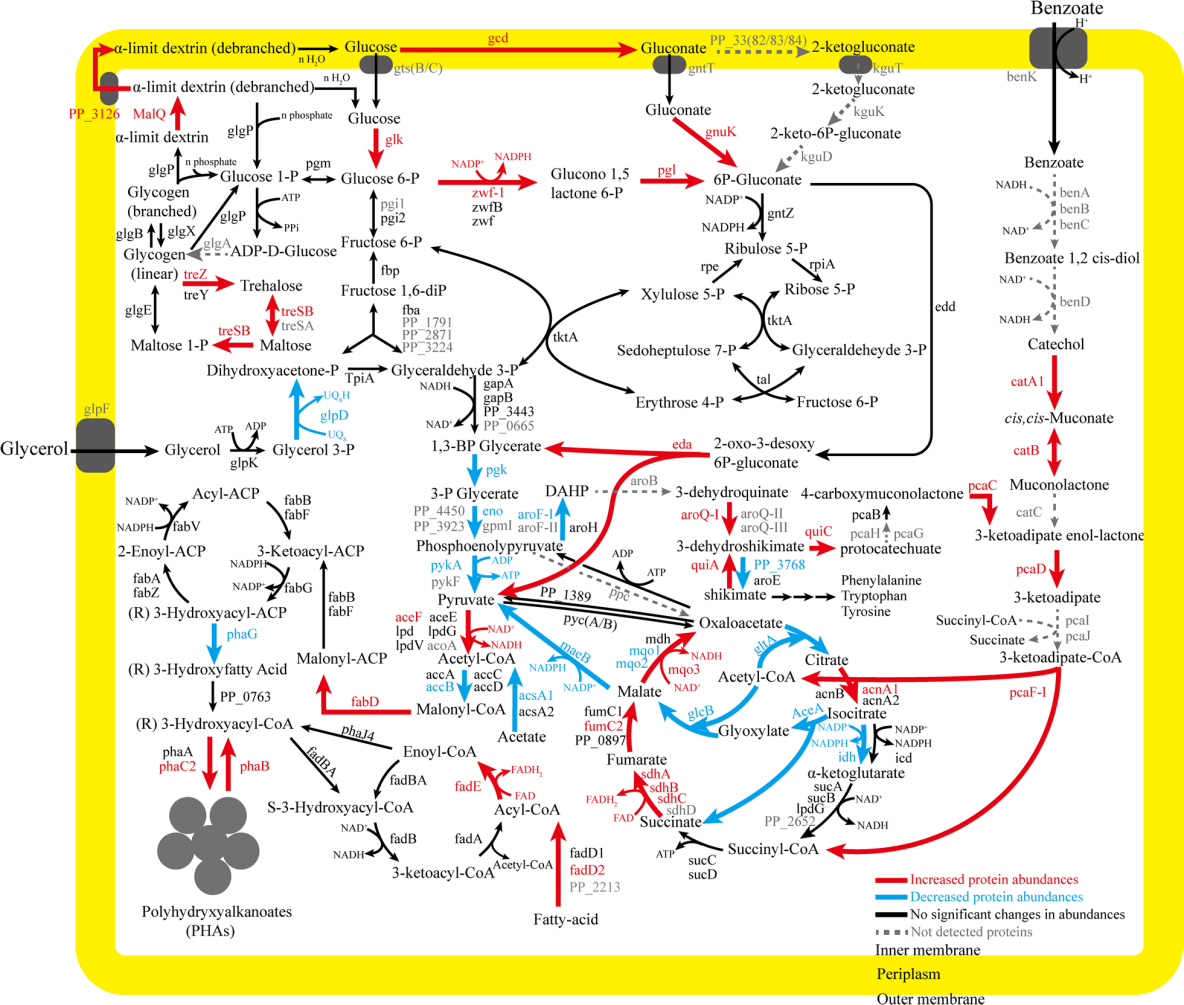
Differential abundance of proteins in *P. putida* KT2440 grown for 3 days in the presence of 1 g L^−1^ benzoate with 9 g L^−1^ glycerol compared to the glycerol control (10 g L^−1^). Increased fold changes of proteins are marked in red, and decreased fold changes of proteins are marked in blue. Non-significant fold changes of proteins are mark in black, and not detected proteins are marked in grey with dashed lines. The abbreviation of each protein is listed in Additional file 3

The top 10 enriched KEGG pathways with a p value less than 0.01 are also presented in descending form in Fig. 5b. Moreover, the overview of the significantly expressed proteins among the enriched KEGG pathways were highlighted in the heatmap afterwards for both up- and down-regulated proteins (Fig. 6a, b) and finally mapped into the metabolic pathway map (Fig. 7). The introduction of benzoate into the carbon source receipt of solo glycerol could act as both a toxic and a nutritional signal, triggering a series of detoxification and degradation systems of the aromatic compound in *P. putida* KT2440. Our results demonstrated that after exposure to the co-feeding conditions of glycerol and benzoate, a precise metabolic response at the level of aromatic degradation pathways was accompanied with a general stress response in different levels, indicated by the induction of enzymes known to respond to oxidative stress and energy generation. Interestingly, the metabolic response appears to reflect a shift of intracellularly available carbon sources towards energy and precursor generation, for instance, which is usually exhibited as a survival strategy to overcome environmental stress in *P. putida* [66–68].

The enriched KEGG pathways revealed that the addition of benzoate significantly impacted the aromatic degradation pathways (Fig. 5b). In Figs. 6a and 7, enzymes involved in the catechol branch of the *β*-ketoadipate pathway [46] showed significant up-regulation. In particular, Catechol 1,2-dioxygenase (catA1) and muconate cycloisomerase 1 (catB) demonstrated a huge, 21.4-fold and 104.3-fold up-regulation, respectively. Besides, the protocatechuate branch [57] was also up-regulated and exhibited lower fold-change compared to the catechol branch, where 4-carboxymuconolactone decarboxylase (pcaC) and 3-oxoadipate enol-lactonase (pacD) demonstrated a 1.4-fold and 1.5-fold up-regulation, respectively. In addition, shikimate degradation, which is connected with the protocatechuate branch, was also up-regulated. For instance, quinate dehydrogenase (quiA) and 3-dehydroshikimate dehydratase (quiC) displayed 1.3-fold, 1.5-fold, and 2.0-fold up-regulation, respectively, suggesting that there were multiple pathways involved in aromatic degradation [69].

Glycerol utilization is negatively regulated by GlpR repressor, which binds to the up-stream binding sites of *glp* clusters such as the *glpF* and *glpD* genes, repressing the glycerol utilization and generating a prolonged lag phase [60]. Glycerol 3-phosphate (G3P) in turn binds to glpR repressor and relieves the glpR-associated repression in a positive feedback loop [70]. Proteomics results indicated that the glpR repressor did not show noteworthy differences in performance under any conditions (Additional file 2: Table S7), suggesting the regulation of *glp* clusters by glpR is not affected by the co-feeding of carbon sources. However, a down-regulation happened to glycerol-3-phosphate dehydrogenase (glpD) under co-feeding conditions (Fig. 6b), resulting in the reduced glycerol assimilation in the presence of benzoate, which was also consistent with previous HPLC results (Figs. 1c and 2c) and metabolite profile (Additional file 1: Figure S5a–c) results.

Notably, consistent with the metabolic profiles results before (Additional file 1: Figure S6a–c), proteomics analysis demonstrated that the addition of benzoate activates the trehalose degradation enzymes, suggesting the participation of trehalose in the energy generation process (Fig. 6a). Trehalose plays a dual role both in osmoregulation and in the metabolism of linear or branched glycogen in *P. putida* KT2440 [71]. Due to lack of the *ostAB* genes that directly connect trehalose with UDP-d-glucose in *P. putida* KT2440, trehalose degradation is by-passed with maltose (bifunctional trehalose synthase B/maltokinase, treSB) and glycogen (Malto-oligosyl trehalose synthase/Malto-oligosyl trehalose trehalohydrolase, treY/treZ) and finally leads to the ED pathway through glucose-1-P (glycogen phosphorylase, glgP). On the other hand, glycogen is also degraded to dextrin through the up-regulated 4-alpha-glucanotransferase (malQ, 1.7-fold), which relates to glycan degradation for generating oligomers or monomers of glucose [72]. Here we also observed an up-regulated polysaccharides transporter (PP_3126, 1.5-fold) which could export the polysaccharides such as dextrin to periplasm space [73], following the hydrolases to produce glucose for further utilization. Consistently, enzymes involved in glucose degradation in peripheral pathways (i.e. direct phosphorylation to glucose 6-phosphate or conversion to gluconate [74, 75]) were up-regulated, including glucose dehydrogenase (gcd), d-gluconate kinase (gnuK), and glucokinase (glk), with 1.5-fold, 1.4-fold and 1.7-fold up-regulation, respectively.

Enzymes involved in the Entner–Doudoroff (ED) pathway were differentially expressed under co-feeding conditions, for instance, Glucose 6-phosphate-1-dehydrogenase (zwf-1), 6-phosphogluconolactonase (pgl), and KHG/KDPG aldolase (eda) were 2.8-fold, 1.8-fold, and 1.7-fold up-regulated, respectively (Fig. 6a, Additional file 2: Table S5). In contrast, several enzymes within the EMP pathway were down-regulated, such as enolase (eno), phosphoglycerate kinase (pgk), and pyruvate kinase II (pykA), indicating that the suppressed EMP pathway and activated ED pathway is more favourable for producing NADPH. During heterotrophic growth on glycerol, the Entner–Doudoroff (ED) pathway and pyruvate metabolism play a key role in PHA biosynthesis according to previous transcriptome and fluxomic analysis of *P. putida* KT2440 [50]. Bhaganna et al. [31] investigated the cellular response and performed the proteomics study with the presence of benzene (5.2 mM) and glycerol (0.52 M) in *P. putida* KT2440. Benzene stress inhibited cell growth, while glycerol protected cell systems by up-regulating glucose-6-phosphate dehydrogenase, isocitrate lyase, and enoyl-CoA hydratase, which are involved in the ED pathway, TCA cycle, and fatty acid beta-oxidation pathway, respectively. Moreover, Chavarria et al. [67] reported that under oxidative stress, the ED pathway was activated for generating the reducing equivalent (NADPH) in *P. putida*. These results are consistent with our findings, which reinforce the hypothesis that the addition of benzoate with glycerol triggers oxidative stress and activates the ED pathway for generating excess reducing equivalents.

Amino acids are the main component of the precursor of biomass synthesis. The catabolism of amino acid is also a necessary process to produce energy under unfavourable growth conditions [76, 77]. However, the enriched biological process and KEGG pathway analysis showed the suppression in both the amino acid biosynthesis and degradation pathways (Fig. 5a, b), indicating that the addition of benzoate not only inhibited the biosynthesis of amino acids, but also refrained from obtaining energy at the expense of the amino acid (l-arginine) degradation process. Alternatively, the reductive tricarboxylic acid cycle, ED pathway, and carbohydrate degradation processes were up-regulated for the generation of energy, in contrast to the down-regulation of the oxidative glyoxylate cycle. Isocitrate dehydrogenase (icd, idh) is a critical branching enzyme between the TCA cycle and glyoxylate shunt, and defends against oxidative damage to the process of generating reducing power (NADPH) [9]. The switch between the TCA cycle and glyoxylate shunt is controlled by isocitrate dehydrogenase kinase/phosphatase (AceK), which activates isocitrate dehydrogenase (icd) by dephosphorylation following the activation of enzymes in the TCA cycle and the inhibition of the glyoxylate shunt, respectively [78]. Isocitrate dehydrogenase kinase/phosphatase (aceK) was up-regulated 1.3-fold, which performing kinase/phosphatase on isocitrate dehydrogenase (icd) and switch off of glyoxylate shunt which was in consistent with the down-regulated proteins such as isocitrate lyase (aceA) and malate synthase G (glcB). Moreover, other enzymes involved in the TCA cycle were also up-regulated, including aconitate hydratase (acnA1, 1.5-fold), succinate dehydrogenase (sdhA, 1.4-fold; sdhB, 1.5-fold; sdhC, 1.7-fold), class 2 fumarate hydratase (fumC2, 1.4-fold), and malate:quinone oxidoreductase (mqo3, 1.3-fold) in *P. putida* KT2440, suggesting the activation of the reductive branch of TCA cycle to produce energy and reducing power such as ATP and NAD(P)H [68]. Previous publication has reported that [78] the abundance of oxaloacetate and pyruvate in *Pseudomonas aeruginosa* are key activators of isocitrate dehydrogenase (idh) and isocitrate dehydrogenase kinase/phosphatase (aceK), which follows the dephosphorylation of isocitrate dehydrogenase (icd), activating the TCA cycle and inhibiting the glyoxylate shunt. Moreover, in the case of co-feeding glucose with benzoate in *P. putida* KT2440, isocitrate lyase (aceA) and isocitrate dehydrogenase (icd) were both down-regulated [5]. Our results were partially consistent with previous reports with regards to the up-regulation of aceK and insignificant changes in icd, whereas other isocitrate dehydrogenase (idh) was down-regulated, indicating that there might be additional mechanisms of isocitrate dehydrogenase (idh) under co-feeding conditions in *P. putida* KT2440.

Some of the enzymes involved in cell wall biosynthesis were down-regulated with the glycerol and benzoate co-feeding conditions (Fig. 6b). For example, *N*-acetyl-β-muramate 6-phosphate phosphatase (mupP), beta-*N*-acetylglucosaminidase (nagZ), glucans biosynthesis protein G (opgG), and glucose-1-phosphate thymidylyltransferas (rfbA), involved in peptidoglycan biosynthesis, were down-regulated. Besides, UDP-3-O-acylglucosamine (lpxD), which participates in lipid A biosynthesis and is located on the surface of the membrane, was also down-regulated. The bacterial cell wall provides structural integrity and plays an important role in regulating cellular envelope balance under stress conditions [79]. The down-regulated cell wall synthesis proteins might lead to cell wall rearrangement or deficiency, redirecting intracellular carbon sources into reducing equivalent generation processes that could help the cell overcome the stress [80].

Bacteria express several enzymes that play roles in the detoxification of reactive oxygen species (ROS) [15]. The proteomics results (Fig. 6a, b) confirmed that *P. putida* KT2440 responds to oxidative stress by up-regulating catalase (katE), catalase–peroxidase (katG), and cytochrome c551 peroxidase (PP_2943) with hydrogen peroxidase alleviating capacity, accompanied by the aromatic degradation process. This data is in agreement with several previous reports that observed a significant abundance of the above-mentioned protein induced when *Pseudomonas* was grown under stress conditions [56, 81, 82]. Besides, the glutathione reduction system was also activated in responding to oxidative stress, for instance, glutathione peroxidase (PP_1686) and glutathionyl hydroquinone reductase (gqr) were up-regulated 1.6- and 2.5-fold, respectively. These enhanced enzymes indicated that the aromatic degradation involved oxidative conditions by generating hydrogen peroxidase, corresponding to the activation of antioxidant systems in *P. putida*, which was widely reported as being in lignin-derived compound degradation in *P. putida* [15, 56, 57]. Conversely, other oxidative stress response enzyme systems, such as thioredoxin (trx, trxA, and trxB), peroxiredoxin (ahpC), and glutathione system (gor, gshB), were down-regulated. Due to the reducing power requirement (e.g. NADH or NADPH) in order to recycle the small antioxidant molecules, the expression of these enzymes might be dynamically altered [56]. The same trend was observed for NADH dehydrogenase (ndh) and NAD(P)H dehydrogenase (PP_1644), showing the interconversion of NAD^+^/NADH and NADP^+^/NADPH was also suppressed.

The fatty acid metabolism was directly correlated with PHA synthesis and being found more active during the co-feeding of glycerol with benzoate (Fig. 6a). The fatty acid *β*-oxidation was up-regulated, which breaks down the fatty acids and produces NADH and acetyl-CoA for the cell to maintain redox homeostasis and more importantly, as the precursor for PHA synthesis. For instance, long-chain fatty acid-CoA ligase (fadD2) and acyl-CoA dehydrogenase (fadE) were up-regulated 1.7-fold and 1.4-fold, respectively. The oxidative stress generated by the aromatic compound degradation might be the reason for inducing the up-regulation of enzymes in fatty acid oxidation, which was consistent with previous publications [13, 35]. In contrast, several enzymes related to fatty acid biosynthesis were down-regulated (Fig. 6a, b). In particular, acetyl-coenzyme A synthetase (acsA1) and the biotin carboxyl carrier protein of acetyl-CoA carboxylase (accB) were down-regulated, which is related to the initial step of acetyl-CoA conversion. However, malonyl-CoA-ACP transacylase (fabD), the rate-limiting step in fatty acid biosynthesis, was up-regulated. Previous studies reported that the up-regulation of fabD enhanced growth by increasing the unsaturated longer chain length of fatty acids, which might be an alternative bacterial strategy to alleviate the n-butanol stress in *E. coli* [83, 84]. Furthermore, enzymes directly involved in PHA synthesis, such as poly(R)-3-hydroxyalkanoate polymerase 2 (phaC2), were up-regulated 1.5-fold, suggesting the enhancement of PHA synthesis under co-feeding conditions. Besides, enzymes linked between PHA biosynthesis and fatty acid synthesis, such as (R)-3-hydroxydecanoyl-ACP: CoA transacylase (phaG), were down-regulated, limiting the fatty acids from biosynthesis process from entering the PHA synthesis pathway. Therefore, our results demonstrated that the monomer composition of PHA is dynamically balanced between fatty acid biosynthesis and *β*-oxidation. The proteomics results reinforced the GC–MS results, where the decreasing of long-chain monomers and increasing of short-chain monomers of the PHA and maintained the unsaturated fatty acid content in co-feeding conditions. In addition, the PHA depolymerizing enzyme, poly(3-hydroxyalkanoate) depolymerase (phaB), was also up-regulated 1.4-fold, indicating that PHA is also dynamically regulated in order to maintain cellular redox balance.

Overall, the mechanism of co-feeding of benzoate and glycerol in *P. putida* KT2440 could possibly involve the following steps (Figs. 6a, b, and 7). First, the addition of benzoate generates oxidative stress and thus influences intracellular redox balance. Then, the redox balance is potentially influenced by the increased NAD^+^/NADH ratio and NADPH/NADP^+^ ratio, corresponding to enhancing the oxidative-stress response reactions (e.g., katE/G and gqr) and energy generation pathways, which includes the ED pathway (e.g. zwf-1, pgl, eda), the reducing TCA route (e.g. sdhA/B/C, fumC2, mqo3), the trehalose degradation pathway (e.g. treSB, treZ), and fatty acid *β*-oxidation pathway (e.g. fadD2, fadE). At last, the excess amount of reducing equivalents (NAD(P)H) is stored as PHA polymer through the up-regulation of PHA polymerase (phaC2). Therefore, the enhancement of PHA content is not due to the increased equivalent carbon and nitrogen ratio, but due to the increased inner reducing power pools (NAD(P)H). Besides, the addition of lignin break-down products may trigger the redox regulation, manipulate the intracellular reducing power elevation, and influence the carbon efficiency under co-feeding conditions, which sheds light on a promising direction towards efficient lignin valorization. Further research efforts can be carried out to understand the balance of energy, reductant, and carbon source related to efficient electron transfer in order to maximize the PHA yield.

## Conclusions

This study revealed that the co-metabolism of glycerol and lignin derivatives (i.e. benzoate, vanillin, and vanillic acid) simultaneously enhanced the cell dry weight and biosynthesis of PHA in *P. putida* KT2440. GC–MS analysis of PHA composition exhibited a decrease in proportion of long-chain monomers (C10 and C12) and an increase in proportion of short-chain monomers (C6 and C8). The two-dimensional HMBC/HSQC/COSY NMR analysis confirmed that the PHA monomers (C6–C14) were produced with glycerol alone or co-feeding of glycerol and benzoate. The effects of benzoate addition on both CDW and PHA content were independent from the nitrogen availability, which ruled out any possible correlation with an increased carbon-to-nitrogen ratio. ^1^H, ^13^C, and ^31^P NMR performed for intracellular and extracellular metabolites plus energetic state analysis revealed that the addition of lignin-derived compounds (e.g. benzoate) affected the supply of reducing power and enhanced the metabolism of glycerol as well as PHA accumulation. Proteomics revealed that the up-regulation of enzymes was involved in aromatic and trehalose degradation, oxidative-stress responses, energy generation (ED pathway and reducing TCA route), and PHA accumulation (fatty acid *β*-oxidation and PHA biosynthesis). These findings provide new insights into significant improvements of carbon efficiency and increasing the yields of biofuels and bioproducts.

## Methods

### Microorganism and medium

*Pseudomonas putida* KT2440 was purchased from American Type Culture Collection (ATCC 47054) and conserved in 25% glycerol at − 80 °C. All the chemical reagents were purchased from Sigma Aldrich and Fisher Scientific with ACS grade (99% purity). The stock culture was pre-streaked in a LB medium plate for 24 h at 28 °C, and then one colony was picked up and inoculated to 200 mL LB liquid medium for 24 h at 28 °C and 180 rpm. The cells were harvested by centrifugation (8014×*g*, 5 min), washed twice with sterilized 0.9% (w/v) NaCl solution, and then suspended with 20 mL sterilized 0.9% (w/v) NaCl solution as seed inoculum. *P. putida* KT2440 cells were used to inoculate the M9 culture medium to an initial OD600 of 0.3 (around 10 mg cell dry weight). The M9 mineral medium contained (per litre): 6 g NaHPO_4_, 3 g KH_2_PO_4_, 0.5 g NaCl, 0.12 g MgSO_4_, 0.72 mg (NH_4_)_6_Mo_7_O_24_·4H_2_O, 24.7 mg H_3_BO_3_, 7.1 mg CoCl_2_·6H_2_O, 2.5 mg CuSO_4_·5H_2_O, 15.8 mg MnCl_2_·4H_2_O, 2.9 mg ZnSO_4_·7H_2_O, 1.4 mg FeSO_4_·7H_2_O, and 11.1 mg CaCl_2_. This M9 basic solution was supplemented with various carbon sources, including glycerol, benzoate, vanillin, and vanillic acid in addition to different concentrations of NH_4_Cl [85]. Cultures were grown in multiple 250-mL Erlenmeyer flasks with 100 mL of defined M9 medium at 28 °C and 180 rpm in batch. Three flasks from each treatment were taken periodically as samples for analysis of cell growth, PHA compositions, substrate concentrations, and metabolite profiling.

### Effects of lignin derivatives on cell growth and PHA compositions

*Pseudomonas putida* KT2440 were fed on 10 g L^−1^ glycerol as the main carbon source with the addition of 0.5 g L^−1^ benzoate, vanillin, or vanillic acid as the co-metabolic substrate, with a fixed NH_4_Cl concentration of 0.8 g L^−1^. The bacterial cells were fed in 100 mL M9 medium in 250-mL flask with the above-mentioned carbon and nitrogen source conditions. Every treatment was conducted in triplicates. The cells were harvested at different time point of fermentation (0 h, 12 h, 24 h, 48 h, 72 h). The cell dry weight, PHA content, glycerol and benzoate utilization, and nitrogen concentration were analysed.

### Effects of the ratio of glycerol to benzoate on cell growth and PHA synthesis

In total six conditions were set for different carbon sources, including glycerol 10 g L^−1^, glycerol 9 g L^−1^ plus benzoate 1 g L^−1^, glycerol 8 g L^−1^ plus benzoate 2 g L^−1^, glycerol 7 g L^−1^ plus benzoate 3 g L^−1^, glycerol 6 g L^−1^ plus benzoate 4 g L^−1^, and glycerol 5 g L^−1^ plus benzoate 5 g L^−1^. The nitrogen source, NH_4_Cl, was set as 0.8 g L^−1^. *P. putida* strains were fed in 100 mL M9 medium in 250-mL flask with above-mentioned carbon and nitrogen source conditions. Every treatment was conducted in triplicates. The cells were harvested at different time point of fermentation (0 h, 24 h, 48 h, 72 h, 96 h, 120 h). The cell dry weight, PHA content, glycerol and benzoate utilization were analysed.

### Effects of nitrogen availability on cell growth and PHA accumulation

The total carbon source remained the same at 10 g L^−1^ with various combinations, including glycerol 10 g L^−1^ (108.6 mM, total C 325.8 mM), glycerol 9 g L^−1^ (97.7 mM) plus benzoate 1 g L^−1^ (8.2 mM, total C 350.5 mM), glycerol 5 g L^−1^ (54.3 mM) plus benzoate 5 g L^−1^ (40.9 mM, total C 449.2 mM), and benzoate 10 g L^−1^ (81.9 mM, total C 573.3 mM). The nitrogen source, NH_4_Cl, also varied in different concentrations, including 0.2 g L^−1^ (3.7 mM), 0.5 g L^−1^ (9.3 mM), 0.8 g L^−1^ (15 mM), and 1.1 g L^−1^ (20.6 mM). *P. putida* was grown in 100 mL M9 medium in 250 mL flask under above-mentioned carbon and nitrogen source conditions. Every treatment was conducted in triplicates. The cells were harvested at 72 h of fermentation. The cell dry weight and PHA content were investigated under different glycerol/benzoate/nitrogen conditions.

### Microbial growth analysis

To determine the cell dry weight (CDW), 100 mL fermentation broth was transferred to 50-mL centrifuge tube and centrifuged for 5 min at 8014×*g*. 2 mL supernatant from each sample was transferred to 2-mL centrifuge tubes and stored at – 20 °C for substrate analysis and nitrogen determination purpose. The cell pellets were washed twice with 0.9% NaCl solution, freeze-dried, and weighed.

### Analysis of soluble compounds in fermentation broth

1 mL stored supernatant was filtered through 0.2 µm nylon filter and stored in HPLC vial for substrate analysis. Concentrations of glycerol, benzoate, vanillin, and vanillic acid in the fermentation broth was quantified by high performance liquid chromatography (HPLC) through an Aminex HPX-87H column at a flow rate of 0.6 mL min^−1^ at 65 °C using 4 mM sulfuric acid as the mobile phase. A refractive index detector was used to analyse glycerol and a photodiode array detector (Dionex ultimate 3000 model) was used for benzoate, vanillin, and vanillic acid [86].

### Determination of nitrogen concentration

The nitrogen concentration during fermentation was measured following previously established methods [25, 87]. The fermentation supernatant samples were diluted and mixed with the following reagents, including 0.4 mL 10% phenol (in ethanol), 0.4 mL 0.5% nitroprusside solution, and 1 mL 20% mixed solution of trisodium citrate and sodium hypochlorite (trisodium citrate with 1% sodium hydroxide mixed with 5% sodium hypochlorite at a ratio of 4:1 (v/v)). All the samples were incubated at 37 ℃ for 2 h and photometrically quantified by measuring colorimetric changes at 630 nm.

### Extraction and characterization of PHA

Methods of PHA polymer extraction and methanolysis were described in details in our previous publication [25]. The freeze-dried cells (around 30 mg) were ground, weighed, and extracted with 7 mL chloroform in a 50-mL glass serum vial (sealed with rubber stopper and 20 mm aluminium crimp seal) at 60 °C in a shaking incubator at 150 rpm for 12 h (note: chloroform is a hazardous solvent [13]). Each extractive was filtered into separate reaction tubes (KIMAX reusable tubes with a PTFE-faced rubber-lined screw cap, catalogue No. #14-930-10B) using a 0.2-μm nylon membrane filter and concentrated to 1 mL by passing nitrogen for about 20 min. 10 mL methanol was added to precipitate the polymer at 4 °C and rest for 3 days. The mixture in methanol was centrifuged at 1127*g* (3000 rpm) for 5 min, separating the organic solution. The methanol–chloroform mixture was treated as chemical waste. The extracted PHA polymers were dried with nitrogen flow for 5 min and then weighed. PHA content and PHA yield were calculated as follows:

1 $$\text{PHA content} (\%) =\frac{\text{weight of extracted PHA polymer (g)}}{{\text{weight of ground cells for extraction (g)} }} \times 100\%,$$

2 $$\text{PHA yield} \;({\text{g}\;\text{L}}^{-1}) =\frac{\text{PHA content } (\%) \times \text{ weight of initial lyophilized cells (g)}}{\text{0.1 L}}.$$

For mcl-PHA methanolysis, PHA polymers produced from each sample were placed in separate close-tightened reaction tubes for further analysis. Methanolysis was carried out by adding 1.7 mL methanol and 0.3 mL concentrated sulfuric acid (final concentration 15% v/v) with PHA polymers and incubating at 100 °C in a conventional oven (BINDER) for 4 h. PHA was broken down into fatty acid methyl esters (FAMEs, indicating as different PHA monomers) in the methanolysis reaction. After cooling, 2 mL dichloromethane was added and vortexed for 1 min to extract the ester component, and then 1 mL Milli-Q water was added to force the organic-phase layering. The bottom dichloromethane layer was transferred into a glass vial, neutralized with 0.20 g CaCO_3_, and then filtered with 0.2 μm nylon membrane into the GC vials. The organic phase was analysed by GC–MS (7890A GC-system with 5975C MSD, Agilent Technologies) equipped with a DB-5 capillary column (30 m × 0.250 μm × 0.25 μm) to identify the monomeric compositions of PHA. 1 μL of the organic phase was injected into the gas chromatograph in splitless mode using helium as the carrier gas at a flow rate of 1.2 mL min^−1^. The oven temperature was programmed at an initial temperature of 40 °C and subsequently raised to 280 °C at a rate of 10 °C min^−1^ and held for 5 min. Positive ions were obtained using electron ionization at 70 eV in full scans. PHA monomers were identified initially through library comparison using the NIST database. A series of diluted (ranging from 10 to 200 µg, calculated based on the weight of standard and the concentration of FAME listed in the manual) FAME standards mix (SUPELCO 37-component FAME mix, #CRM47885) were analysed together with the PHA samples to confirm the PHA monomer identification. The external standard curve was established for identified PHA monomers (indicated as FAMEs ranging from C6 to C14) based on the peak area in each concentration of the standard sample. The weight of each monomer in each PHA sample was calculated based on the specific standard curve. The percentage of PHA monomers was calculated as the weight of specific monomer divided by the sum of total weight of all monomers in each PHA sample [25].

### NMR analysis for PHA characterization

Two-dimensional (2D) ^1^H-correlation spectroscopy (^1^H-COSY), heteronuclear single quantum correlation (HSQC) and heteronuclear multiple bond correlation (HMBC) were performed on a Bruker Avance HD III 500 MHz spectrometer. The detailed acquisition parameters were listed in the previous publication [25]. 30 mg of isolated PHA sample or 30 mg of cell samples containing PHA was dissolved in 0.5 mL CDCl_3_ for NMR analysis. For the cell samples, the NMR sample solutions were sonicated for 4 h. The COSY, HSQC and HMBC experiments were carried out with standard Bruker sequences “cosyetgp”, “hsqcetgpsisp2.2” and “hmbccgpndqf”, respectively, with a scan number of 16 for COSY and of 96 for HSQC and HMBC. All the NMR spectra were analysed with the software Mestrenova (version of 12.0.2) [88].

### Metabolic products analysis using NMR spectroscopy

To prepare samples of extracellular metabolites for NMR spectroscopy analysis, 6 mL of fermentation broth was centrifuged at 8,000 rpm for 5 min at 4 °C. The resulting supernatant was freeze-dried. To determine intracellular metabolites, the fermentation broth was centrifuged, and then around 0.24 g wet cell pellet was mixed with ice-cold chloroform/methanol (2:1, v/v) solution and subjected to three cycles of freezing/thawing using liquid nitrogen. Following centrifugation, the sample separated into three layers (i.e. aqueous layer, cell debris, and organic layer). The entire upper layer (aqueous MeOH) was removed and evaporated with nitrogen flow. For each sample, the dried extract from cells and lyophilized powder from supernatant was mixed with 600 μL of D_2_O solution containing 0.5 mM DSS-d6 (2,2 dimethyl 2-silapentane-d6 5-sulfonate, sodium salt) and 0.2% (w/v) sodium azide as a microbiocide [89]. Extracellular and intracellular metabolite samples were placed in 5 mm NMR tubes. All the NMR spectra were collected at 25 °C on a Bruker Avance HD III 500-MHz NMR system equipped with a cryoprobe. The ^13^C and ^31^P NMR experiments were operated at the frequency of 125.77 MHz for the ^13^C nucleus and 202.49 MHz for ^31^P nucleus, respectively. NMR spectra were obtained using standard Bruker pulse sequence (zgig) with an inverse-gated WALTZ16 proton decoupling pulse for both ^13^C and ^31^P NMR experiments. The solvent peak of DSS (δc 0.0 ppm) was used for ^13^C chemical shifts calibration. The reference for ^31^P chemical shift (^31^P δ 140.0 ppm) was measured with an external sample of trimethyl phosphite (1% in deuterated benzene).

### NADH and NADPH quantification

Four combinations of different carbon sources were used in the experiments, including glycerol 10 g L^−1^, glycerol 9.5 g L^−1^ plus benzoate 0.5 g L^−1^, glycerol 9 g L^−1^ plus benzoate 1 g L^−1^, glycerol 8 g L^−1^ plus benzoate 2 g L^−1^. The nitrogen source was set as 0.8 g L^−1^. *P. putida* grew in 100 mL M9 medium in a 250-mL flask with above-mentioned carbon and nitrogen sources. Every treatment was conducted in triplicates. Around 0.1 mL crude cells were harvested at different time point of fermentation (12 h, 24 h, 48 h, 72 h). Crude cell lysates were obtained by undertaking three freeze–thaw (− 80 °C and 30 °C) lysis cycles. Concentrations of intracellular NAD^+^, NADH, NADP^+^ and NADPH were quantified with a NADP^+^/NADPH and NAD^+^/NADH Colorimetric Quantitation Kit (Biovision, Abcam, UK) and colorimetric changes at 450 nm were measured [90].

### LC–MS/MS label-free proteomics analysis

*P. putida* grew in 100 mL M9 medium in a 250-mL flask with 10 g L^−1^ glycerol or 9 g L^−1^ glycerol + 1 g L^−1^ benzoate as the carbon source in an incubator shaker at 180 rpm and 28 °C (*n* = 3 for each condition). After 72 h fermentation, cell pellets and supernatant were separated by centrifugation at 8014×*g*, 4 °C for 15 min. Cell pellets were then washed twice with 0.9% (w/v) NaCl solution and resuspended in lysis buffer (8 M urea, 75 mM NaCl in 100 mM NH_4_HCO_3_, pH 7.8), followed by 8 rounds of 30 s bead beating using a Bullet Blender (Homogenizers, Atkinson, NH) [91]. Lysate was collected and centrifuged at 10,000 rpm, 4 °C for 10 min to remove cellular debris. The supernatant was concentrated using 30-kDa filter (EMD Millipore, Billerica, MA) by centrifugation at 4000 rpm, 4 °C for 30 min. The concentrated supernatant was transferred to a clean tube for further digestion. The protein purification and digestion of all samples were conducted with the FASP Protein Digestion Kit (Expedeon, San Diego, CA) with trypsin (Promega, Madison, WI) following the manufacturer’s instruction. The protein concentration was estimated by the Pierce™ BCA protein assay (Thermo Scientific, San Jose, CA) and normalized to 0.1 μg μL^−1^ before LC–MS/MS analysis. Biological triplicates were applied during the entire process.

LC–MS/MS analysis was performed using a Q-Exactive HF Mass Spectrometer (Thermo Scientific, San Jose, CA). Data were acquired for 100 min when the gradient started. The detailed equipment parameters setup was described in a previous publication by Cao et al. [92]. In terms of proteomic data analysis, raw MS/MS data files were processed with MaxQuant (version 1.6.7.0). After loading all the raw data and given appropriate names. Label-free quantification (LFQ) algorithm was used with minimum LFQ ratio count of 2 for relative quantification in the Group-specific parameters section. Trypsin was selected for digestion mode with the maximum of two missed cleavages. The peptide tandem mass spec raw data were searched against the Uniport FASTA files of strain *P. putida* KT2440 (released at 07, April 2017, Taxonomy ID: 160488). In global parameters section, the second peptides and match between runs features were enabled with a 0.7-min match time window and 20-min alignment time window. The spectral level false discovery rate (FDR, q value) was <  = 1% based on a decoy search [93]. Other parameters just followed the default settings. The protein intensities obtained from Maxquant software were log2 transformed and then subjected to Perseus software (version 1.6.12.0) for statistical analysis. ANOVA tests with permutation-based FDR calculation and Student’s t-test were conducted to determine the statistical significance between glycerol control and co-feeding treatment. Statistically significant proteins were defined using strict criteria of both q value < 0.05 and *p*-value < 0.05 as cutoff values. A total of 1926 proteins were quantified, including 249 up-regulated proteins and 214 down-regulated proteins under glycerol and benzoate co-feeding conditions, compared to the glycerol control (q value < 0.05 and fold changes > 1.3). The list of up-regulated and down-regulated proteins (Additional file 2: Tables S5, S6) was first merged and then subjected to gene ontology (GO) enrichment analysis. Biological process (BP) and KEGG pathway enrichment analyses were performed to compare the bacterial physiological and metabolic responses to glycerol and co-feeding glycerol + benzoate conditions. Gene ontology (GO) analysis was performed with BioCyc pathway/Genome Database Collection website (BioCyc, biocyc.org). The significant proteins were separated into up- or down-regulated groups based on Student’s t-test differences (positive or negative values). The uniport IDs for those two groups of protein were first converted to gene names in Uniport database and then uploaded to Biocyc database as new smart tables, respectively. The enrichment analysis for biological process (BP) was performed with *p*-value < 0.01 as the cutoff value and Fisher Exact Parent–Child Intersection as the statistical method [94, 95]. The enrichment analysis for KEGG pathway enrichment analysis was performed with *p*-value < 0.01 as the cutoff value and Fisher Exact as the statistical method [95]. The *p*-value for each enriched biological process was − log10 transformed, sorted and plotted from smallest to the largest in Origin software (version 9.6.0.172). The multi experiment viewer software (Mev, version 4.0) [35] was used to generate the heatmap for those proteins showed on average an increase > 1.3-fold to compare the difference of protein expression of culture on glycerol or co-feeding glycerol with benzoate as the carbon source.

## Supplementary Information

**Additional file 1: Figure S1.** GC–MS spectra of PHA monomers from the 72 h’ sample under different carbon feeding strategies. **Figure S2.** Polyhydroxyalkanoate (PHA) chemical structure**. Figure S3.** HMBC spectra of PHA polymer of glycerol 10 g L^−1^ (a) and glycerol 9.5 g L^−1^ + Benzoate 0.5 g L^−1^ at 72 h (b). **Figure S4.** COSY (a) and HSQC (b) spectra of PHA polymer of glycerol 10 g L^−1^ at 72 h. **Figure S5.** ^1^H NMR spectra of fermentation supernatant at 72 h with mixed or solo carbon sources. **Figure S6.** ^1^H NMR spectra of intracellular extracts of *P. putida* KT2440 at 72 h with mixed or solo carbon sources. **Figure S7.** ^13^C NMR spectra of intracellular extracts of *P. putida* KT2440 at 72 h with mixed or solo carbon sources. **Figure S8.** ^31^P NMR spectra of intracellular extracts of *P. putida* KT2440 at 72 h with mixed or solo carbon sources. **Table S1.** Carbon numbers of PHA monomers in HMBC NMR. **Table S2.** Detailed chemical shifts (ppm) from COSY/HSQC/HMBC NMR spectra of PHA monomers. **Table S3.** ^1^H, ^13^C, and ^31^P chemical shifts of referenced compounds, retrieved from the Human Metabolome Database (HMDB) [96] or recorded at 500 MHz in D_2_O at 25 °C. **Table S4.** Intracellular NAD(P)H and NAD(P)^+^ contents (µmol·g_CDW_^−1^) with glycerol and benzoate co-metabolism.

**Additional file 2: Table S5.** Normalized data of up-regulated protein of co-feeding glycerol + benzoate. **Table S6.** Normalized data of down-regulated protein of co-feeding glycerol + benzoate. **Table S7.** Normalized data of all intracellular protein of co-feeding glycerol + benzoate.

**Additional file 3.** A list of abbreviations is included.

## Data Availability

All data generated or analysed during this study are included in this published article and its supplementary information files.
