# Supplementary material for: Enhancement of polyhydroxyalkanoate production by co-feeding lignin derivatives with glycerol in Pseudomonas putida KT2440
Source: Biotechnol Biofuels. 2021 Jan 7;14:11. doi: 10.1186/s13068-020-01861-2 (PMC7792162; doi:10.1186/s13068-020-01861-2)
Supplement: Supplementary file 1 — Additional file 1: Figure S1. GC–MS spectra of PHA monomers from the 72 h’ sample under different carbon feeding strategies. Figure S2. Polyhydroxyalkanoate (PHA) chemical structure. Figure S3. HMBC spectra of PHA polymer of glycerol 10 g L−1 (a) and glycerol 9.5 g L−1 + Benzoate 0.5 g L−1 at 72 h (b). Figure S4. COSY (a) and HSQC (b) spectra of PHA polymer of glycerol 10 g L−1 at 72 h. Figure S5. 1H NMR spectra of fermentation supernatant at 72 h with mixed or solo carbon sources. Figure S6. 1H NMR spectra of intracellular extracts of P. putida KT2440 at 72 h with mixed or solo carbon sources. Figure S7. 13C NMR spectra of intracellular extracts of P. putida KT2440 at 72 h with mixed or solo carbon sources. Figure S8. 31P NMR spectra of intracellular extracts of P. putida KT2440 at 72 h with mixed or solo carbon sources. Table S1. Carbon numbers of PHA monomers in HMBC NMR. Table S2. Detailed chemical shifts (ppm) from COSY/HSQC/HMBC NMR spectra of PHA monomers. Table S3. 1H, 13C, and 31P chemical shifts of referenced compounds, retrieved from the Human Metabolome Database (HMDB) [96] or recorded at 500 MHz in D2O at 25 °C. Table S4. Intracellular NAD(P)H and NAD(P)+ contents (µmol·gCDW−1) with glycerol and benzoate co-metabolism. [file 13068_2020_1861_MOESM1_ESM.docx]

Enhancement of Polyhydroxyalkanoate Production by Co-feeding Lignin Derivatives with Glycerol in *Pseudomonas putida* KT2440

Zhangyang Xu^a^, Chunmei Pan^a,f^, Xiaolu Li^a^, Naijia Hao^b^, Tong Zhang^c^, Matthew J. Gaffrey^c^, Yunqiao Pu^d^, John R. Cort^c^, Arthur J. Ragauskas^b,d,e^, Wei-Jun Qian^c^, and Bin Yang^a,c*^

1. Bioproducts, Sciences & Engineering Laboratory, Department of Biological Systems

Engineering, Washington State University, Richland, Washington, 99354, United States.

1. Department of Chemical and Biomolecular Engineering, University of Tennessee,

Knoxville, Tennessee, 37996, United States.

1. Biological Sciences Division, Pacific Northwest National Laboratory, Richland, Washington, 99352, United States.
2. Joint Institute Biological for Sciences, Biosciences Division, Oak Ridge National Laboratory, Oak Ridge, Tennessee, 37831, United States.
3. Department of Forestry, Wildlife, and Fisheries, Center for Renewable Carbon, University of Tennessee Institute of Agriculture, Knoxville, Tennessee, 37996, United States.
4. College of Food and Bioengineering, Henan University of Animal Husbandry and

Economy, Zhengzhou, Henan, 450046, China.

**Additional Figures and Tables**


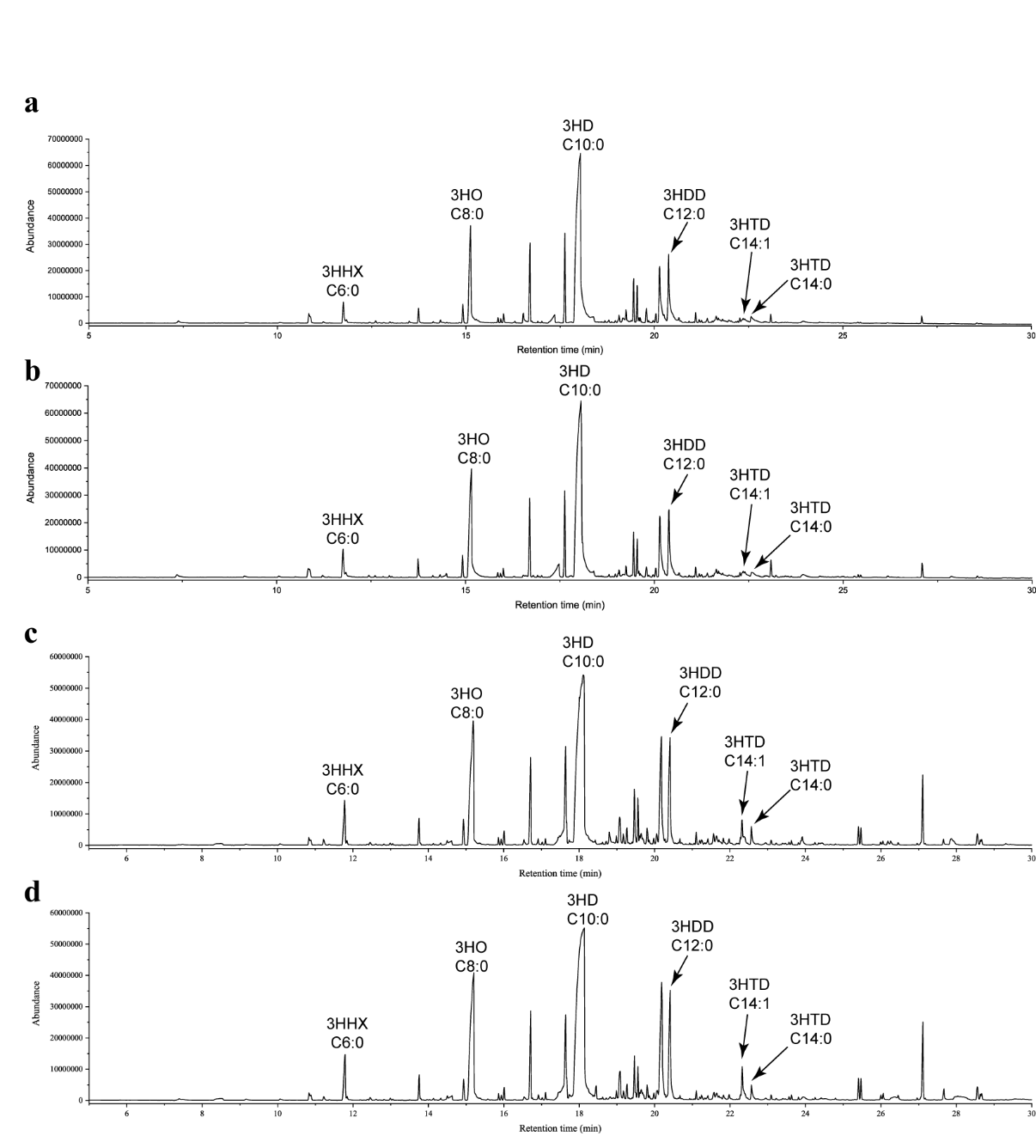


**Figure S1.** GC-MS spectra of PHA monomers from the 72 h’ sample under different carbon feeding strategies. (a) Glycerol 10 g·L^-1^ (b) Glycerol 9.5 g·L^-1^ + Benzoate 0.5 g·L^-1^ (c) Glycerol 9.5 g·L^-1^ + Vanillin 0.5 g·L^-1^ and (d) Glycerol 9.5 g·L^-1^ + Vanillic acid 0.5 g·L^-1^. 3HHX (C6:0) 3-hyxroxyhexanoate, 3HO (C8:0) 3-hyxroxyoctanoate, 3HD (C10:0) 3-hydroxydecanoate, 3HDD (C12:0) 3-hydroxydodecanoate, 3HTD (C14:1, C14:0) 3-hydroxytetradecanoate.


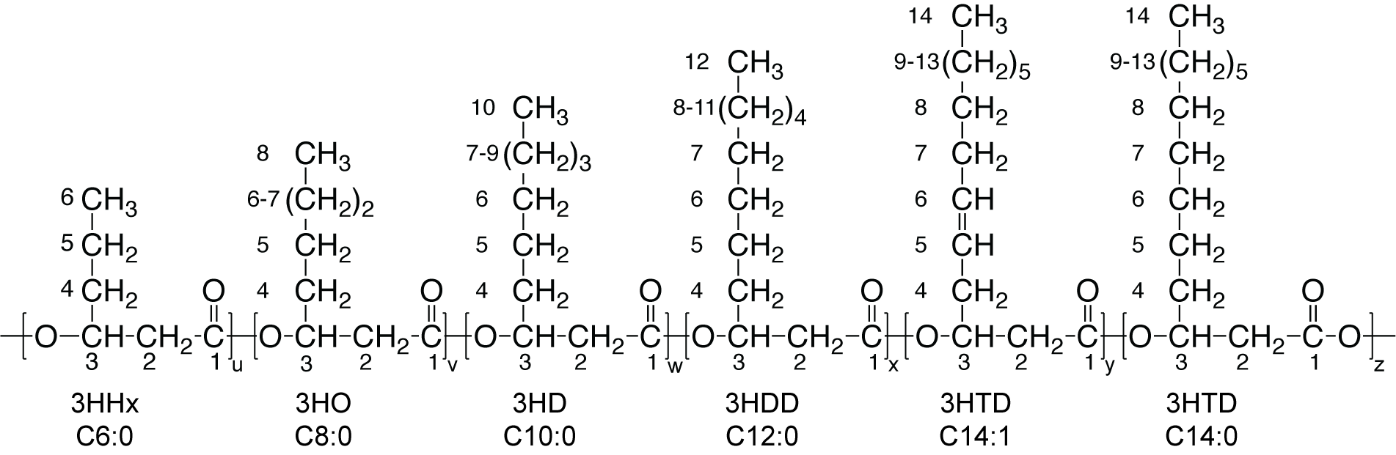


**Figure S2.** Polyhydroxyalkanoate (PHA) chemical structure. 3HHX (C6:0) 3-hyxroxyhexanoate, 3HO (C8:0) 3-hyxroxyoctanoate, 3HD (C10:0) 3-hydroxydecanoate, 3HDD (C12:0) 3-hydroxydodecanoate, 3HTD (C14:1, C14:0) 3-hydroxytetradecanoate. The structural repeating units (SRU) are labeled with lower-case letters (from u to z).


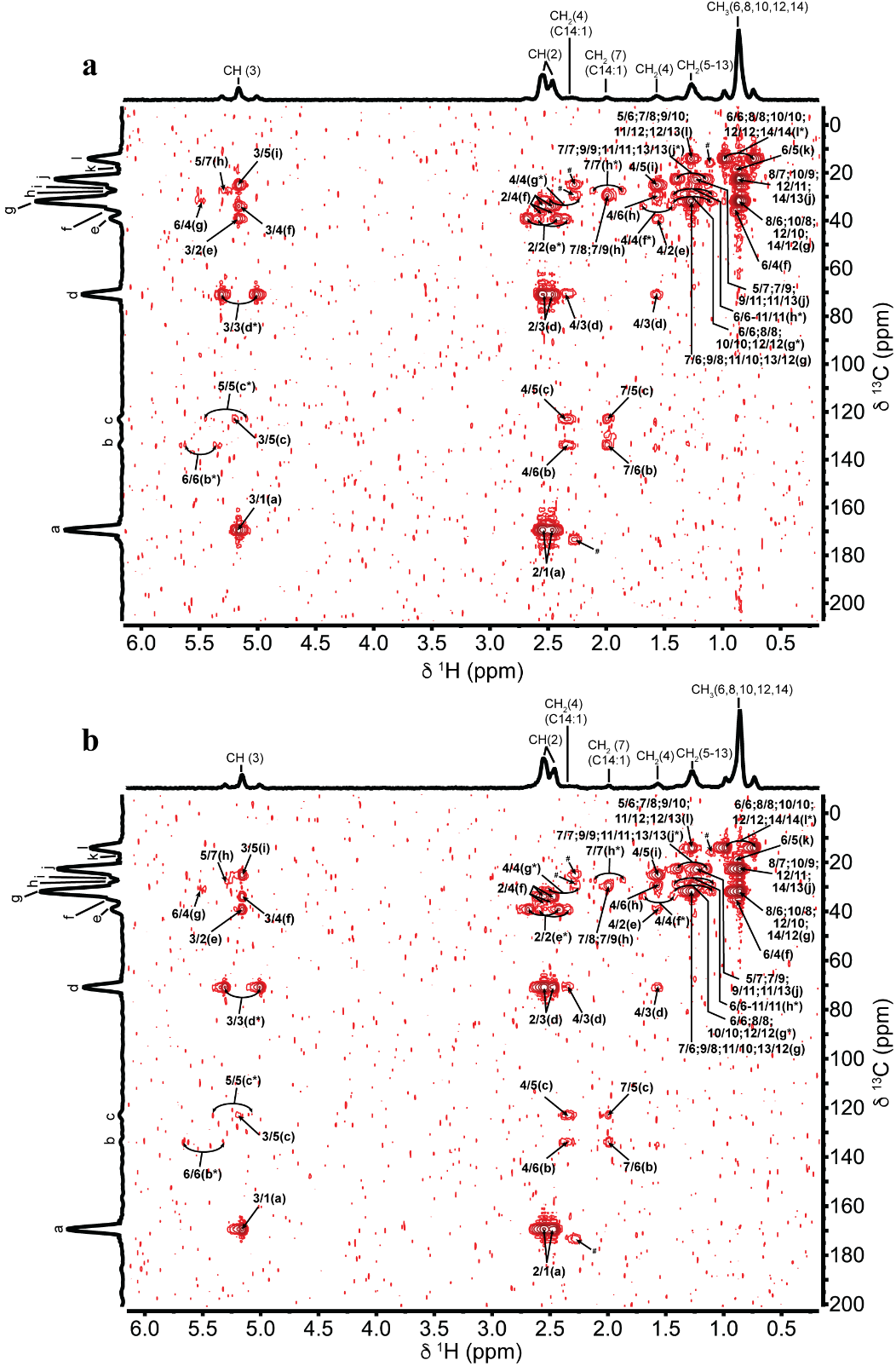


**Figure S3.** HMBC spectra (CDCl_3_ solvent) of PHA polymer from *P. putida* grown on glycerol 10 g·L^-1^ (a) and glycerol 9.5 g·L^-1^ +benzoate 0.5 g·L^-1^ at 72h (b) (two numbers are used to indicate the proton and carbon in the single/multiple bond coherences. For example, 2/1 indicates the correlation between H-2 and C-1). The specific carbon number of each PHA monomer was indicated in parentheses with the lower-case letter and further listed in detail in Table S1. The residual one-bond proton-carbon correlations (doublets) are labeled with an asterisk (*). The unassigned peaks are labeled with a pound (#).


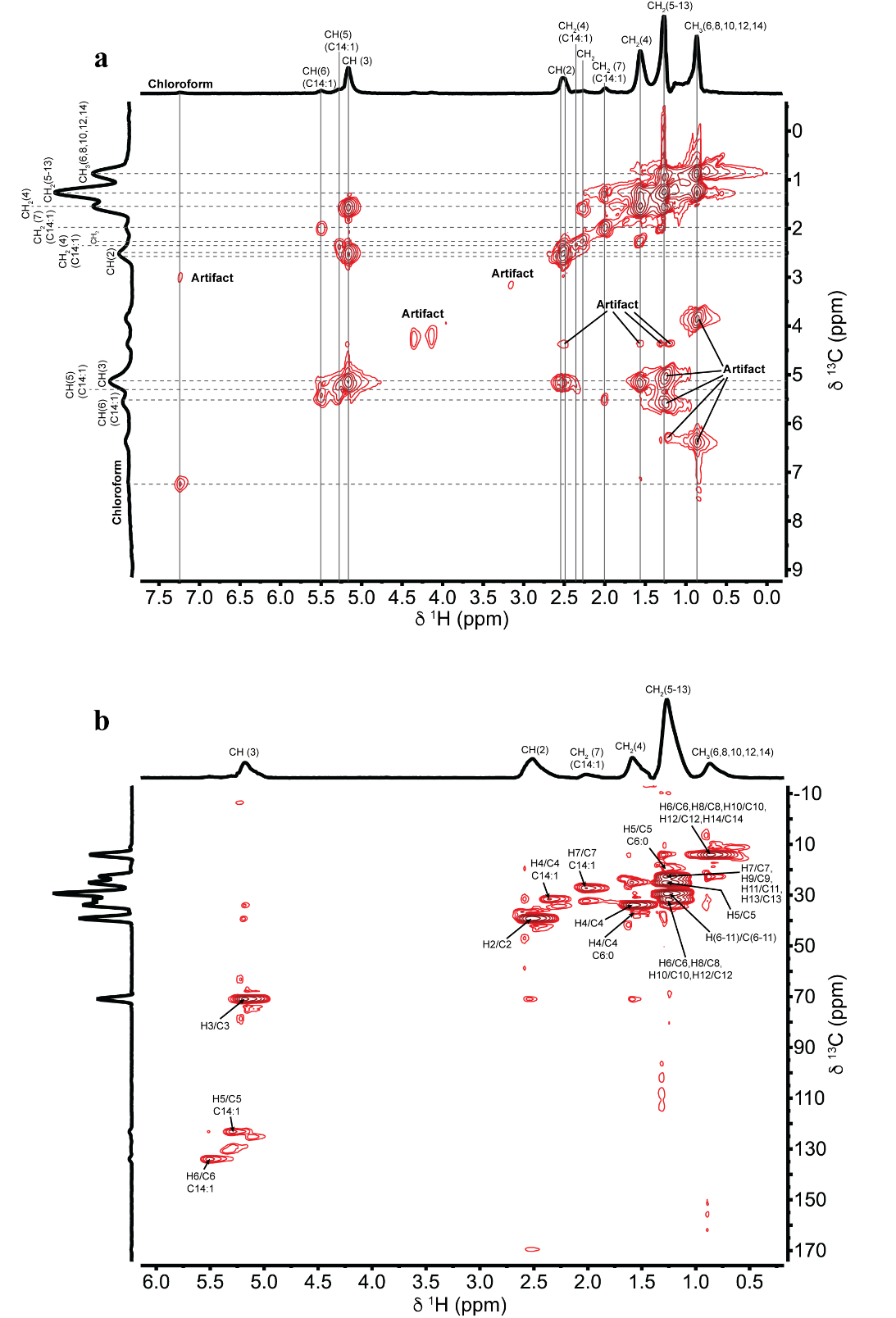


**Figure S4.** COSY (a) and HSQC (b) spectra (CDCl_3_ solvent) of PHA polymer from *P. putida* grown on glycerol 10 g·L^-1^ at 72h (two numbers are used to indicate the proton and carbon in the single bond couplings).


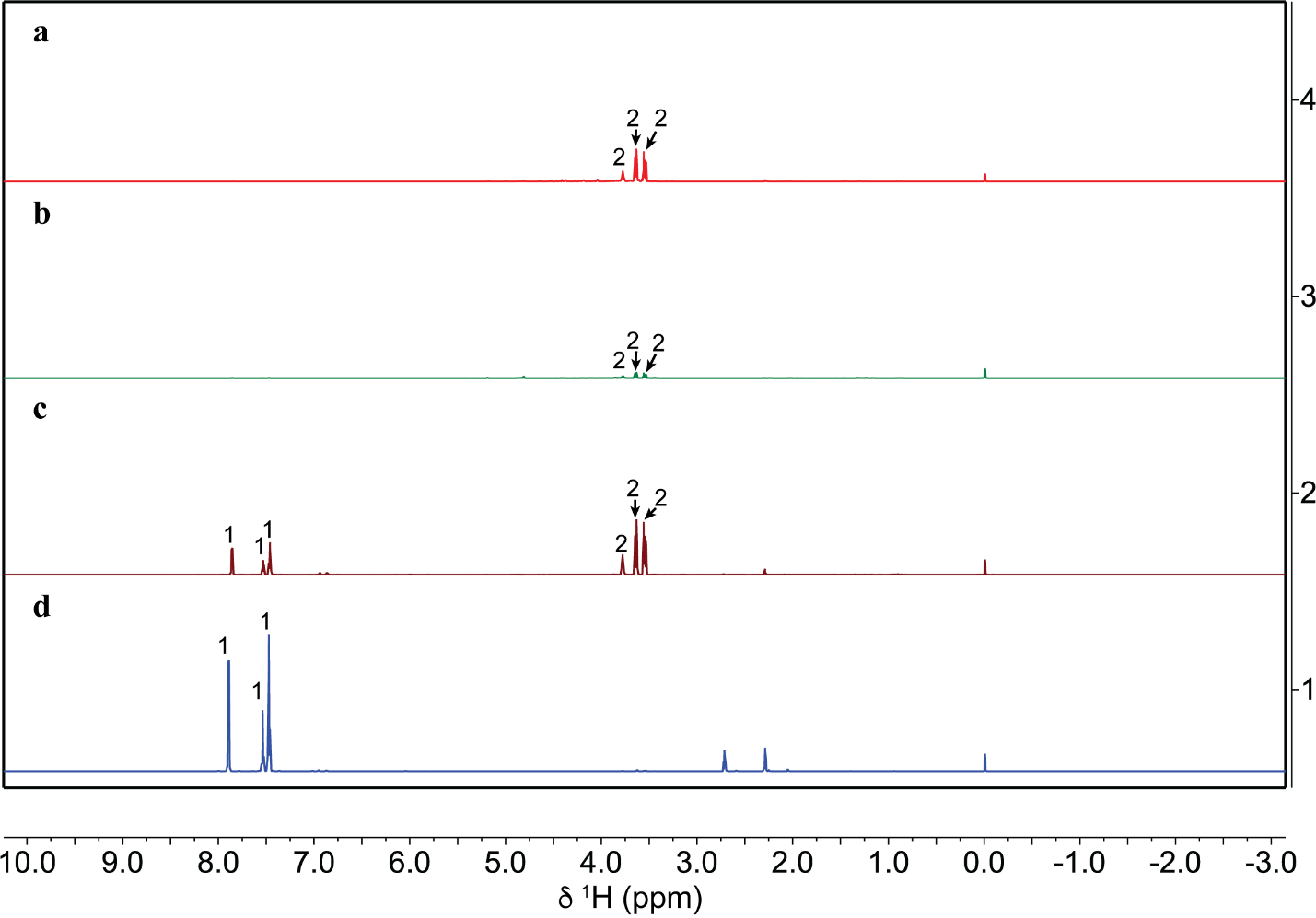


**Figure S5.** ^1^H NMR spectra of fermentation supernatant at 72 h with mixed or solo carbon sources. (a) 10 g/L glycerol; (b) 9 g/L glycerol and 1 g/L benzoate; (c) 5 g/L glycerol and 5 g/L benzoate; (d) 10 g/L benzoate. Each compound was labeled with numbers: 1) benzoate; 2) glycerol.


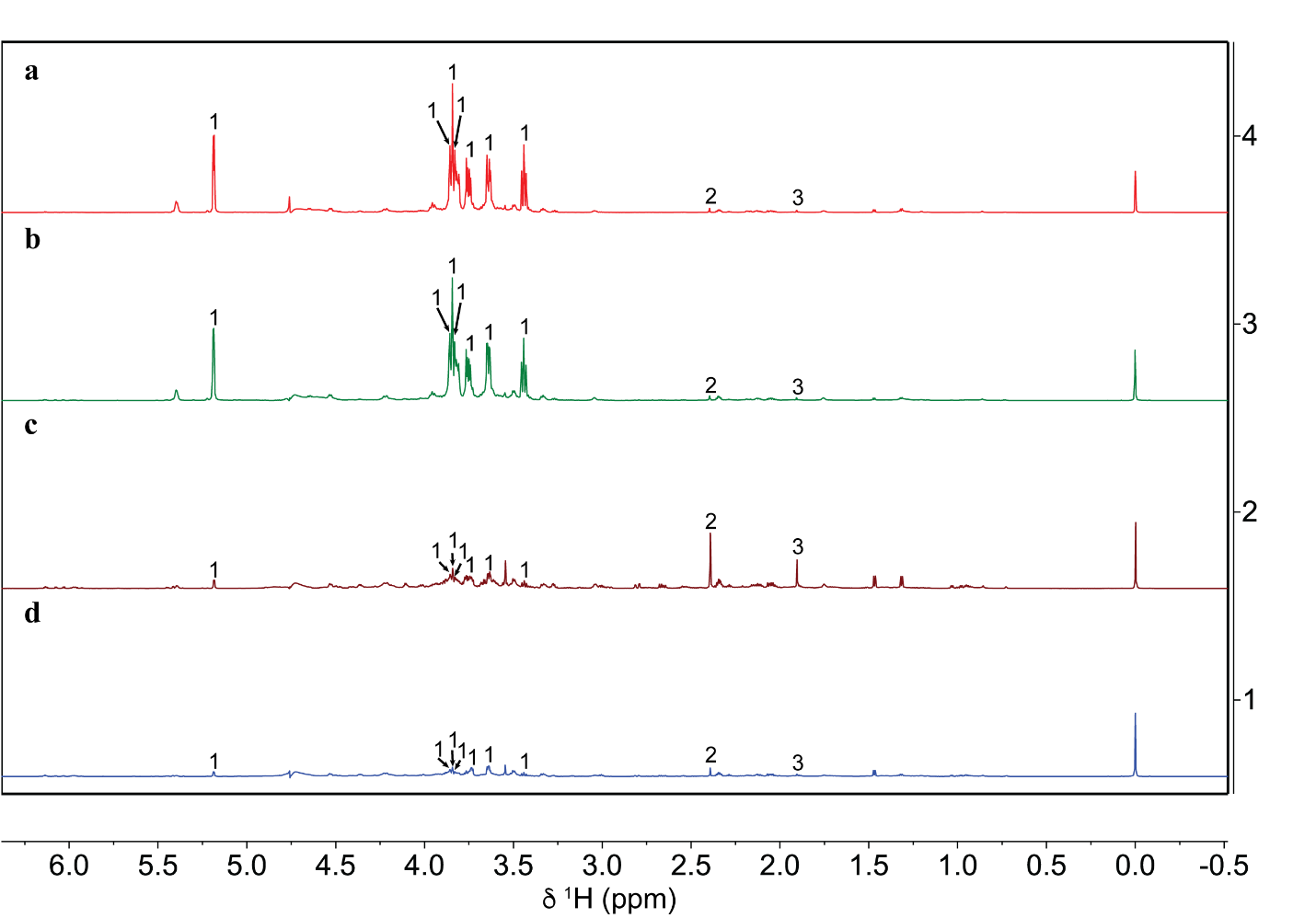


**Figure S6.** ^1^H NMR spectra of intracellular extracts of *P. putida* KT2440 at 72 h with mixed or solo carbon sources. (a) 10 g/L glycerol; (b) 9 g/L glycerol and 1 g/L benzoate; (c) 5 g/L glycerol and 5 g/L benzoate; (d) 10 g/L benzoate. Each compound was labeled with numbers, including 1) trehalose; 2) succinate; 3) acetate.


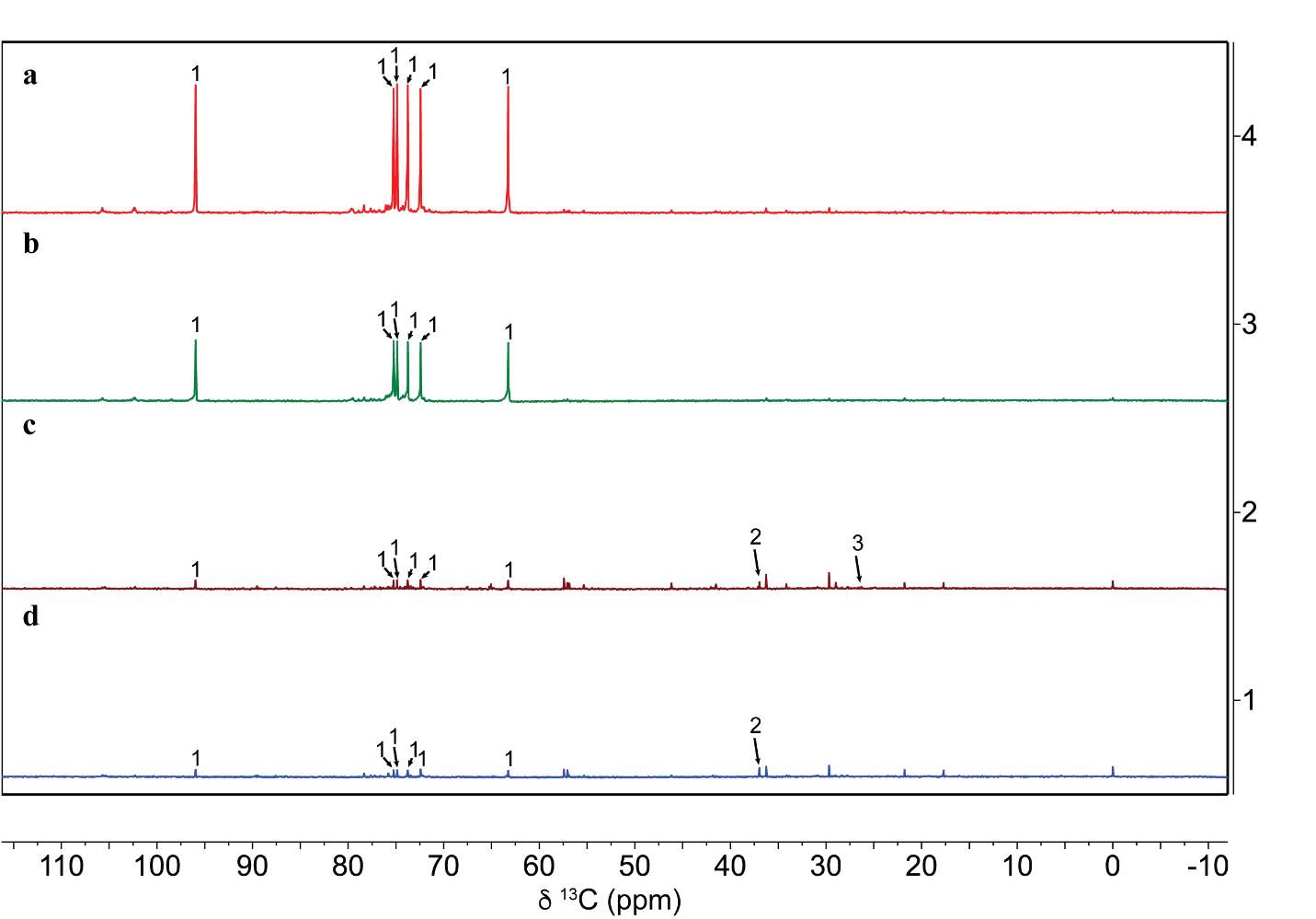


**Figure S7.** ^13^C NMR spectra of intracellular extracts of *P. putida* KT2440 at 72 h with mixed or solo carbon sources. (a) 10 g/L glycerol; (b) 9 g/L glycerol and 1 g/L benzoate; (c) 5 g/L glycerol and 5 g/L benzoate; (d) 10 g/L benzoate. Each compound was labeled with numbers, including 1) trehalose; 2) succinate; 3) acetate.


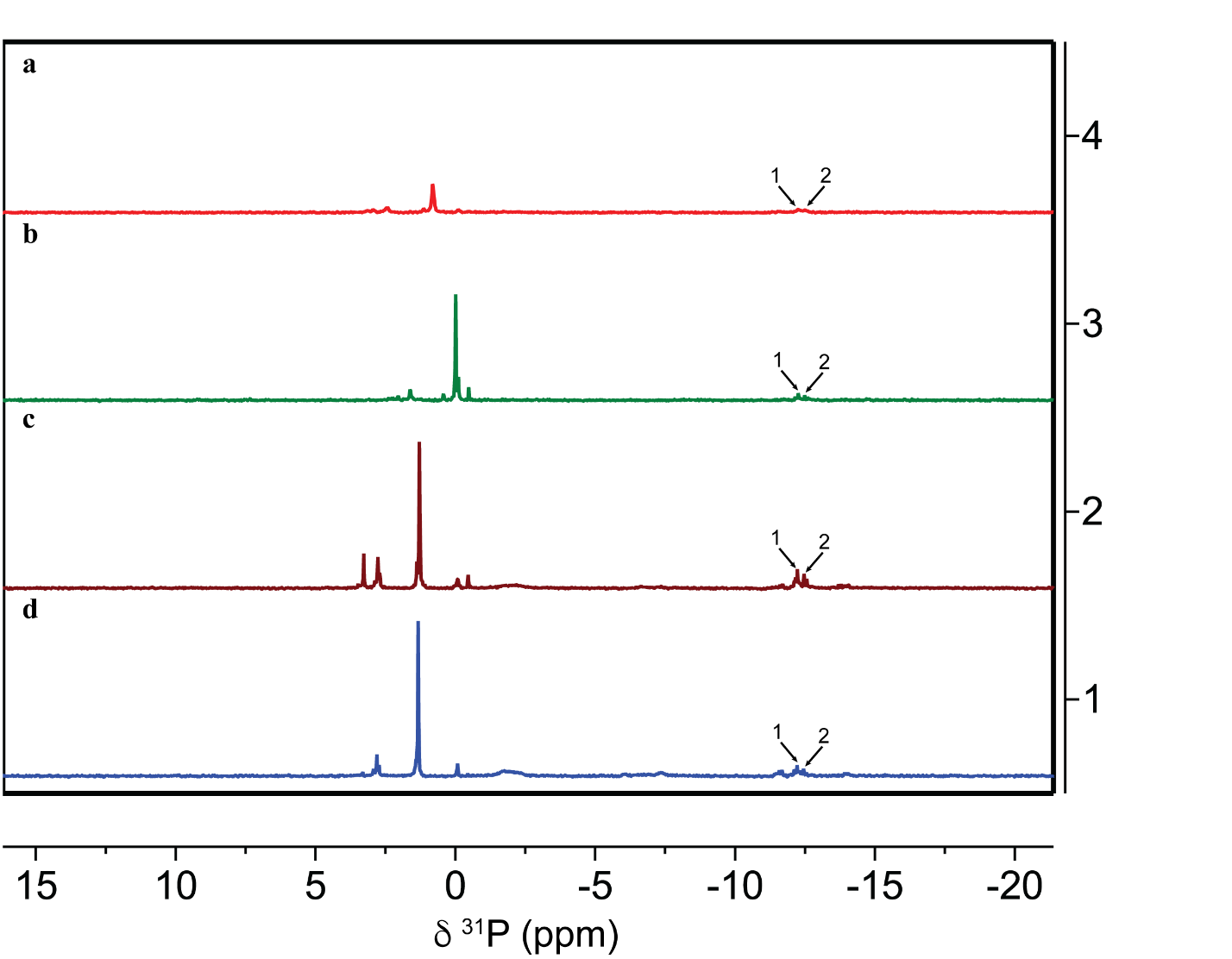


**Figure S8.** ^31^P NMR spectra of intracellular extracts of *P. putida* KT2440 at 72 h with mixed or solo carbon sources. (a) 10 g/L glycerol; (b) 9 g/L glycerol and 1 g/L benzoate; (c) 5 g/L glycerol and 5 g/L benzoate; (d) 10 g/L benzoate. Each compound was labeled with numbers, including 1) NADP^+^; 2) NADPH.

**Table S1.** Carbon numbers of PHA monomers in HMBC NMR.

| **Carbon numbers of 3-hydroxyalkanoate monomers** | | | | | | |
| --- | --- | --- | --- | --- | --- | --- |
| **No.** | **3HHx** | **3HO** | **3HD** | **3HDD** | **3HTD** | **3HTD** |
|  | **C6** | **C8** | **C10** | **C12** | **C14:1** | **C14** |
| a | 1 | 1 | 1 | 1 | 1 | 1 |
| b | - | - | - | - | 6 | - |
| c | - | - | - | - | 5 | - |
| d | 3 | 3 | 3 | 3 | 3 | 3 |
| e | 2 | 2 | 2 | 2 | 2 | 2 |
| f | 4 | 4 | 4 | 4 | - | 4 |
| g | - | 6 | 8 | 10 | 4,12 | 12 |
| h | - | - | 6,7 | 6-9 | 7-11 | 6-11 |
| i | - | 5 | 5 | 5 | - | 5 |
| j | - | 7 | 9 | 11 | 13 | 13 |
| k | 5 | - | - | - | - | - |
| l | 6 | 8 | 10 | 12 | 14 | 14 |

**Table S2.** Detailed chemical shifts (ppm) from COSY/HSQC/HMBC NMR spectra of PHA monomers ^a^.

| **No.** | **3-hydroxyalkanoate monomers (ppm)** | | | | | | | | | | | |
| --- | --- | --- | --- | --- | --- | --- | --- | --- | --- | --- | --- | --- |
|  | **3HHx (C6)** | | **3HO (C8)** | | **3HD (C10)** | | **3HDD (C12)** | | **3HTD (C14:1)** | | **3HTD (C14:0)** | |
|  | **^1^H** | **^13^C** | **^1^H** | **^13^C** | **^1^H** | **^13^C** | **^1^H** | **^13^C** | **^1^H** | **^13^C** | **^1^H** | **^13^C** |
| 1 | - | 169.4 | - | 169.4 | - | 169.4 | - | 169.4 | - | 169.4 | - | 169.4 |
| 2 | 2.46/2.55 | 39.1 | 2.46/2.55 | 39.1 | 2.46/2.55 | 39.1 | 2.46/2.55 | 39.1 | 2.46/2.55 | 39.1 | 2.46/2.55 | 39.1 |
| 3 | 5.16 | 70.9 | 5.16 | 70.9 | 5.16 | 70.9 | 5.16 | 70.9 | 5.16 | 70.9 | 5.16 | 70.9 |
| 4 | 1.57 | 36.5 | 1.57 | 33.8 | 1.57 | 33.8 | 1.57 | 33.8 | 2.35 | 31.2 | 1.57 | 33.8 |
| 5 | 1.26 | 19.3 | 1.23 | 25.1 | 1.27 | 25.1 | 1.27 | 25.1 | 5.30 | 122.9 | 1.27 | 25.1 |
| 6 | 0.86 | 13.8 | 1.26 | 31.7 | 1.26 | 29.3 | 1.26 | 29.3 | 5.50 | 133.9 | 1.26 | 29.3 |
| 7 |  |  | 1.27 | 22.6 | 1.23 | 29.1 | 1.26 | 29.5 | 2.00 | 27.3 | 1.26 | 29.5 |
| 8 |  |  | 0.86 | 13.9 | 1.26 | 31.7 | 1.26 | 29.3 | 1.26 | 29.5 | 1.26 | 29.3 |
| 9 |  |  |  |  | 1.27 | 22.6 | 1.23 | 29.1 | 1.26 | 29.3 | 1.26 | 29.3 |
| 10 |  |  |  |  | 0.86 | 14.0 | 1.26 | 31.7 | 1.26 | 29.3 | 1.26 | 29.3 |
| 11 |  |  |  |  |  |  | 1.27 | 22.6 | 1.23 | 29.1 | 1.23 | 29.1 |
| 12 |  |  |  |  |  |  | 0.86 | 14.0 | 1.26 | 31.7 | 1.26 | 31.7 |
| 13 |  |  |  |  |  |  |  |  | 1.27 | 22.6 | 1.27 | 22.6 |
| 14 |  |  |  |  |  |  |  |  | 0.86 | 14.0 | 0.86 | 14.0 |

^a^ 3-hydroxyhexanoate (C6:0, 3HHX), 3-hydroxyoctanoate (C8:0, 3HO), 3-hydroxydecanoate (C10:0, 3HD), 3-hydroxydodecanoate (C12:0, 3HDD), 3-hydroxytetradecanoate (C14:0/C14:1, 3HTD). ^1^H and ^13^C chemical shifts of PHA monomers are assigned based on COSY/HSQC/HMBC spectra and also referenced to previous publications [1-3]. Overlapping peaks are assigned to the same value, actual values may vary.

**Table S3.** ^1^H, ^13^C, and ^31^P chemical shifts of referenced compounds, retrieved from the Human Metabolome Database (HMDB) or recorded at 500 MHz in D_2_O at 25 °C.

| Compound | Solvent | Atom | ^1^H | ^13^C | ^31^P |
| --- | --- | --- | --- | --- | --- |
| Glycerol^‡^ | D_2_O | 1,3 | 3.64 | 65.3 | - |
|  |  | 1,3 | 3.55 | 65.3 | - |
|  |  | 2 | 3.77 | 74.8 | - |
| Benzoate^‡^ | D_2_O | 2,6 | 7.87 | 131.5 | - |
|  |  | 4 | 7.54 | 133.9 | - |
|  |  | 3,5 | 7.47 | 131.0 | - |
| Trehalose^‡^ | D_2_O | 1,1' | 5.18 | 95.9 | - |
|  |  | 2,2' | 3.63 | 73.8 | - |
|  |  | 3,3' | 3.84 | 75.3 | - |
|  |  | 4,4' | 3.44 | 72.4 | - |
|  |  | 5,5' | 3.80 | 74.8 | - |
|  |  | 6,6' | 3.75 | 63.3 | - |
|  |  | 6,6' | 3.86 | 63.3 | - |
| Succinate^‡^ | D_2_O | 2,3 | 2.38 | 36.8 | - |
| Acetate^‡^ | D_2_O | 1 | 1.90 | 26.0 | - |
| NADP^+^ | D_2_O | - | - | - | -12.24 |
| NADPH | D_2_O | - | - | - | -12.48 |

^‡^Chemical shifts for glycerol, benzoate, trehalose, succinate, and acetate are from HMDB [4]. Atom orders of each compound are based on IUPAC rules.

**Table S4.** Intracellular NAD(P)H and NAD(P)^+^ contents (µmol·g_CDW_^-1^) with glycerol and benzoate co-metabolism (n=3).

| Substrate | 12 h | | 24 h | | 48 h | | 72 h | |
| --- | --- | --- | --- | --- | --- | --- | --- | --- |
|  | NADH | NADPH | NADH | NADPH | NADH | NADPH | NADH | NADPH |
| glycerol 10 g·L^-1^ | 0.13±0.03 | 0.049±0.005 | 0.82±0.07 | 0.13±0.02 | 0.90±0.09 | 0.092±0.004 | 0.34±0.04 | 0.15±0.01 |
| glycerol 9.5g·L^-1^ + Benzoate 0.5g·L^-1^ | 0.10±0.01 | 0.035±0.003 | 0.57±0.05 | 0.082±0.006 | 0.72±0.05 | 0.10±0.007 | 0.31±0.02 | 0.23±0.01 |
| glycerol 9 g·L^-1^ + Benzoate 1 g·L^-1^ | 0.12±0.02 | 0.04±0.002 | 0.51±0.02 | 0.088±0.004 | 0.74±0.06 | 0.15±0.03 | 0.32±0.03 | 0.16±0.03 |
| glycerol 8 g·L^-1^ + Benzoate 2 g·L^-1^ | 0.16±0.01 | 0.053±0.009 | 0.85±0.06 | 0.16±0.01 | 1.10±0.1 | 0.29±0.05 | 0.35±0.03 | 0.12±0.02 |

**References**

1. Tan G-YA, Chen C-L, Li L, Ge L, Wang L, Razaad IM, Li Y, Zhao L, Mo Y, Wang J-Y: **Start a Research on Biopolymer Polyhydroxyalkanoate (PHA): A Review**. *Polymers* 2014, **6**(3).

2. de Waard P, van der Wal H, Huijberts GN, Eggink G: **Heteronuclear NMR analysis of unsaturated fatty acids in poly(3-hydroxyalkanoates). Study of beta-oxidation in Pseudomonas putida**. *J Biol Chem* 1993, **268**(1):315-319.

3. Sathiyanarayanan G, Bhatia SK, Song H-S, Jeon J-M, Kim J, Lee YK, Kim Y-G, Yang Y-H: **Production and characterization of medium-chain-length polyhydroxyalkanoate copolymer from Arctic psychrotrophic bacterium Pseudomonas sp. PAMC 28620**. *International Journal of Biological Macromolecules* 2017, **97**:710-720.

4. Wishart DS, Feunang YD, Marcu A, Guo AC, Liang K, Vázquez-Fresno R, Sajed T, Johnson D, Li C, Karu N *et al*: **HMDB 4.0: the human metabolome database for 2018**. *Nucleic acids research* 2018, **46**(D1):D608-D617.
