# Supplementary material for: Enhancement of polyhydroxyalkanoate production by co-feeding lignin derivatives with glycerol in Pseudomonas putida KT2440
Source: Biotechnol Biofuels. 2021 Jan 7;14:11. doi: 10.1186/s13068-020-01861-2 (PMC7792162; doi:10.1186/s13068-020-01861-2)
Supplement: Supplementary file 3 — Additional file 3. A list of abbreviations is included. [file 13068_2020_1861_MOESM3_ESM.docx]

**Abbreviations**

| **Abbreviation** | **Protein/metabolite name** |
| --- | --- |
| accA | Acetyl-coenzyme A carboxylase carboxyl transferase subunit alpha |
| accB | Biotin carboxyl carrier protein of acetyl-CoA carboxylase |
| accC | Biotin carboxylase |
| accD | Acetyl-coenzyme A carboxylase carboxyl transferase subunit beta |
| aceA | Isocitrate lyase |
| aceE | Pyruvate dehydrogenase |
| aceF | Pyruvate dehydrogenase E2 component |
| aceK | Isocitrate dehydrogenase kinase/phosphatase |
| acnA1 | Aconitate hydratase |
| acnA2 | Aconitate hydratase |
| acnB | Aconitate hydratase B |
| acoA | Acetoin:2,6-dichlorophenolindophenol oxidoreductase subunit alpha |
| acsA1 | Acyl-CoA synthetase |
| acsA2 | Acetyl-coenzyme A synthetase 2 |
| ahpC | peroxiredoxin/alkyl hydroperoxide reductase C |
| aldB-II | Aldehyde dehydrogenase (pedI) |
| aroB | 3-dehydroquinate synthase |
| aroE | Shikimate 5-dehydrogenase |
| aroF-I | Phospho-2-dehydro-3-deoxyheptonate aldolase |
| aroF-II | Phospho-2-dehydro-3-deoxyheptonate aldolase |
| aroH | Phospho-2-dehydro-3-deoxyheptonate aldolase |
| aroQ-I | type II 3-dehydroquinate dehydratase |
| aroQ-II | type II 3-dehydroquinate dehydratase |
| aroQ-III | type II 3-dehydroquinate dehydratase |
| benA | Benzoate 1,2-dioxygenase subunit alpha |
| benB | Benzoate 1,2-dioxygenase subunit beta |
| benC | Benzoate 1,2-dioxygenase electron transfer component |
| benD | 1,6-dihydroxycyclohexa-2,4-diene-1-carboxylate dehydrogenase |
| benK | Benzoate MFS transporter |
| catA1 | Catechol 1,2-dioxygenase |
| catB | Muconate cycloisomerase 1 |
| catC | Muconolactone Delta-isomerase |
| cheA | Chemotaxis histidine kinase CheA |
| cti | Esterified fatty acid cis/trans isomerase |
| curA | NADPH-dependent curcumin/dihydrocurcumin reductase |
| DAHP | 3-Deoxy-D-arabino-heptulosonate-7-phosphate |
| eda | KHG/KDPG aldolase |
| edd | Phosphogluconate dehydratase |
| eno | Enolase |
| etfA | Electron transfer flavoprotein subunit alpha |
| etfB | Electron transfer flavoprotein subunit beta |
| fabA | 3-hydroxydecanoyl-ACP dehydratase |
| fabB | 3-oxoacyl-ACP synthase 1 |
| fabD | Malonyl CoA-ACP transacylase |
| fabF | 3-oxoacyl-ACP synthase 2 |
| fabG | 3-oxoacyl-ACP reductase |
| fabV | Enoyl-ACP reductase [NADH] |
| fabZ | 3-hydroxyacyl-ACP dehydratase FabZ |
| fadA | 3-ketoacyl-CoA thiolase |
| fadB | Fatty acid oxidation complex subunit alpha |
| fadBA | 3-hydroxyacyl-CoA dehydrogenase type-2 |
| fadD1 | Long-chain-fatty-acid/CoA ligase |
| fadD2 | Long-chain-fatty-acid/CoA ligase |
| fadE | Acyl-CoA dehydrogenase |
| fba | Fructose-1,6-bisphosphate aldolase |
| fbp | Fructose-1,6-bisphosphatase class 1 |
| fumC1 | class 2 fumarate hydratase |
| fumC2 | class 2 fumarate hydratase |
| gapA | Glyceraldehyde-3-phosphate dehydrogenase |
| gapB | Glyceraldehyde-3-phosphate dehydrogenase |
| gcd | Quinoprotein glucose dehydrogenase |
| glcB | Malate synthase G |
| glgA | Glycogen synthase |
| glgB | 1,4-alpha-glucan branching enzyme |
| glgE | Alpha-1,4-glucan:maltose-1-phosphate maltosyltransferase |
| glgP | Glycogen phosphorylase |
| glgX | Glycogen debranching enzyme |
| glk | Glucokinase |
| glpD | Glycerol-3-phosphate dehydrogenase |
| glpF | Aquaglyceroporin |
| glpK | Glycerol kinase |
| gltA | Citrate synthase |
| gntT | D-gluconate transporter |
| gntZ | 6-phosphogluconate dehydrogenase |
| gnuK | D-gluconate kinase |
| gor | Glutathione reductase |
| gpmI | 2,3-bisphosphoglycerate-independent phosphoglycerate mutase |
| gqr | Glutathionyl-hydroquinone reductase |
| gshB | Glutathione synthetase |
| gtsB | Mannose/glucose ABC transporter, permease protein |
| gtsC | Mannose/glucose ABC transporter, permease protein |
| hbdH | 3-hydroxybutyrate dehydrogenase |
| icd | Isocitrate dehydrogenase [NADP] |
| idh | Isocitrate dehydrogenase [NADP] |
| katE | Catalase |
| katG | Catalase-peroxidase |
| kguD | Phosphonate dehydrogenase |
| kguK | 2-ketogluconokinase |
| kguT | 2-ketogluconate transporter, putative |
| lpd | Dihydrolipoyl dehydrogenase |
| lpdG | Dihydrolipoyl dehydrogenase |
| lpdV | Dihydrolipoyl dehydrogenase |
| lpxD | UDP-3-O-acylglucosamine N-acyltransferase |
| maeB | Malic enzyme B |
| malQ | 4-alpha-glucanotransferase |
| mdh | Malate dehydrogenase |
| mmsB | 3-hydroxyisobutyrate dehydrogenase |
| mqo1 | Malate:quinone oxidoreductase |
| mqo2 | Malate:quinone oxidoreductase |
| mqo3 | Malate:quinone oxidoreductase |
| mupP | *N*-acetyl-β-muramate 6-phosphate phosphatase |
| murD | UDP-N-acetylmuramoylalanine--D-glutamate ligase |
| nagZ | Beta-N-acetylglucosaminidase |
| ndh | NADH dehydrogenase |
| opgG | Glucans biosynthesis protein G |
| pcaB | 3-carboxy-cis,cis-muconate cycloisomerase |
| pcaC | 4-carboxymuconolactone decarboxylase |
| pcaD | 3-oxoadipate enol-lactonase |
| pcaF-I | Beta-ketoadipyl-CoA thiolase |
| pcaG | protocatechuate 3,4-dioxygenase subunit alpha |
| pcaH | protocatechuate 3,4-dioxygenase subunit beta |
| pcaI | 3-oxoadipate CoA-transferase subunit A |
| pcaJ | 3-oxoadipate CoA-transferase subunit B |
| pgi1 | Glucose-6-phosphate isomerase 1 |
| pgi2 | Glucose-6-phosphate isomerase 2 |
| pgk | Phosphoglycerate kinase |
| pgl | 6-phosphogluconolactonase |
| pgm | Phosphoglucomutase |
| phaA | Poly(3-hydroxyalkanoate) polymerase 1 |
| phaB | Poly(3-hydroxyalkanoate) depolymerase |
| phaC2 | Poly(R)-3-hydroxyalkanoate polymerase 2 |
| phaG | (R)-3-hydroxydecanoyl-ACP:CoA transacylase |
| phaJ4 | (R)-specific enoyl-CoA hydratase |
| PP_0370 | Acyl-CoA dehydrogenase family protein |
| PP_0665 | Glyceraldehyde-3-phosphate dehydrogenase |
| PP_0897 | Fumarate hydratase class I |
| PP_1389 | Oxaloacetate decarboxylase |
| PP_1644 | NAD(P)H dehydrogenase (Quinone) |
| PP_1686 | Glutathione peroxidase |
| PP_1720 | Alcohol dehydrogenase |
| PP_1791 | Putative Aldolase/synthase |
| PP_1816 | Alcohol dehydrogenase |
| PP_2871 | class II aldolase/adducin domain-containing protein |
| PP_3126 | Putative polysaccharide exported protein |
| PP_3224 | class II aldolase/adducin domain-containing protein |
| PP_3382 | Gluconate 2-dehydrogenase cytochrome c subunit |
| PP_3383 | Gluconate 2-dehydrogenase flavoprotein subunit |
| PP_3384 | Gluconate 2-dehydrogenase gamma subunit |
| PP_3443 | Glyceraldehyde-3-phosphate dehydrogenase |
| PP_3768 | Putative shikimate 5-dehydrogenase |
| PP_3923 | Phosphoglycerate mutase |
| PP_4450 | Phosphoglycerate mutase family protein |
| PP_4975 | Long-chain acyl-CoA thioester hydrolase family protein |
| PP_2943 | cytochrome c551 peroxidase |
| ppc | Phosphoenolpyruvate carboxylase |
| pycA | Pyruvate carboxylase subunit A |
| pycB | Pyruvate carboxylase subunit B |
| pykA | Pyruvate kinase II |
| pykF | Pyruvate kinase |
| quiA | Quinate dehydrogenase (Quinone) |
| quiC | 3-dehydroshikimate dehydratase |
| rfbA | Glucose-1-phosphate thymidylyltransferase |
| rffG | dTDP-glucose 4,6-dehydratase |
| rpe | Ribulose-phosphate 3-epimerase |
| rpiA | Ribose-5-phosphate isomerase A |
| sdhA | succinate dehydrogenase flavoprotein subunit |
| sdhB | succinate dehydrogenase iron-sulfur subunit |
| sdhC | succinate dehydrogenase membrane b-556 subunit |
| sdhD | succinate dehydrogenase hydrophobic membrane anchor subunit |
| sucA | 2-oxoglutarate decarboxylase, thiamine-requiring E1 subunit |
| sucB | 2-oxoglutarate dehydrogenase dihydrolipoyltranssuccinylase subunit |
| sucC | succinyl-CoA synthetase subunit beta |
| sucD | succinyl-CoA synthetase subunit alpha |
| tal | Transaldolase |
| tktA | Transketolase |
| tpiA | Triosephosphate isomerase |
| treSB | bifunctional trehalose synthase B/maltokinase |
| treY | Malto-oligosyl trehalose synthase |
| treZ | Malto-oligosyl trehalose trehalohydrolase |
| trx | Thioredoxin |
| trxA | Thioredoxin |
| trxB | Thioredoxin reductase |
| zwf | Glucose 6-phosphate-1-dehydrogenase |
| zwf-1 | Glucose 6-phosphate-1-dehydrogenase |
| zwfB | Glucose 6-phosphate-1-dehydrogenase |
|  |  |
